# A New *Ciboria* sp. for Soil Mycoremediation and the Bacterial Contribution to the Depletion of Total Petroleum Hydrocarbons

**DOI:** 10.3389/fmicb.2021.647373

**Published:** 2021-06-08

**Authors:** Simone Becarelli, Ilaria Chicca, Salvatore La China, Giovanna Siracusa, Alessandra Bardi, Maria Gullo, Giulio Petroni, David Bernard Levin, Simona Di Gregorio

**Affiliations:** ^1^Department of Biology, University of Pisa, Pisa, Italy; ^2^BD Biodigressioni, Pisa, Italy; ^3^Department of Biosystem Engineering, University of Manitoba, Winnipeg, MB, Canada; ^4^Department of Life Sciences, University of Modena and Reggio-Emilia, Reggio Emilia, Italy; ^5^Department of Civil and Environmental Engineering, University of Florence, Florence, Italy

**Keywords:** *Ciboria* sp., dye-decolorizing peroxidases DyP, generalist bacterial species, specialist bacterial species, total petroleum hydrocarbons, mycoremediation, functional metagenomic prediction

## Abstract

A *Ciboria* sp. strain (Phylum Ascomycota) was isolated from hydrocarbon-polluted soil of an abandoned oil refinery in Italy. The strain was able to utilize diesel oil as a sole carbon source for growth. Laboratory-scale experiments were designed to evaluate the use of this fungal strain for treatment of the polluted soil. The concentration of total petroleum hydrocarbons (TPH) in the soil was 8,538 mg/kg. Mesocosms containing the contaminated soil were inoculated with the fungal strain at 1 or 7%, on a fresh weight base ratio. After 90 days of incubation, the depletion of TPH contamination was of 78% with the 1% inoculant, and 99% with the 7% inoculant. 16S rDNA and ITS metabarcoding of the bacterial and fungal communities was performed in order to evaluate the potential synergism between fungi and bacteria in the bioremediation process. The functional metagenomic prediction indicated *Arthrobacter*, *Dietzia*, *Brachybacerium*, *Brevibacterium*, *Gordonia*, *Leucobacter*, *Lysobacter*, and *Agrobacterium* spp. as generalist saprophytes, essential for the onset of hydrocarbonoclastic specialist bacterial species, identified as *Streptomyces*, *Nocardoides*, *Pseudonocardia*, *Solirubrobacter*, *Parvibaculum*, *Rhodanobacter*, *Luteiomonas*, *Planomicrobium*, and *Bacillus* spp., involved in the TPH depletion. The fungal metabolism accelerated the onset of specialist over generalist bacteria. The capacity of the *Ciboria* sp. to deplete TPH in the soil in treatment was also ascertained.

## Introduction

Pollution in soils and sediments is primarily due to total petroleum hydrocarbons (TPH) (Van Liedekerke et al., 2014). Their presence in environmental matrices is due to activities of the chemical processing industry, leaks from underground hydrocarbon storage tanks, and accidental spills. The aromatic fraction of TPH is responsible for their recalcitrance to biodegradation (Booth et al., 2008). The selective pressure exerted by these contaminants leads to the adaptation of microorganisms capable of utilizing TPH components as carbon sources, resulting in the transformation, degradation, and eventually mineralization of these compounds (Margesin et al., 2003; Ron and Rosenberg, 2014; Lea-Smith et al., 2015; Xu et al., 2018). Microorganisms are able to both transform these toxic compounds as well as utilize them as energy sources, individually or under co-metabolic conditions, or they can decrease the toxicant bioavailability and stabilize them in the environment (Peng et al., 2008; Ghosal et al., 2016).

A large number of bacterial and fungal species and strains that are able to transform TPH have been described. Fungi have been described as very competitive in disturbed soils, because they can spread in the environment via hyphae elongation, and adopt growth strategies to resist to physical stresses, such as lack of nutrients and water (Harms et al., 2011). Fungal hyphal growth is combined with the secretion of extracellular polyphenols oxidases and laccases that normally contribute to the transformation of macromolecules recalcitrant to biodegradation, such as lignin and soil organic matter (Harms et al., 2011; Marco-Urrea et al., 2015). The extracellular battery of enzymes can be exploited also for the transformation of xenobiotic molecules, recalcitrant to biodegradation (Baldrian, 2004; Schmidt et al., 2005). Bacteria too have been described as able to produce polyphenols oxidases capable to transform lignin and soil organic matter (de Gonzalo et al., 2016).

Microbial polyphenol oxidases and laccases generate reactive oxygen species, removing electrons from polyphenolic macromolecules, to yield radical cation intermediates, resulting in aromatic ring opening and the breakdown of polyphenolic and polyaromatic compounds. On the other hand, the same radical cation intermediates are subjected to polycondensation reactions (Harms et al., 2011). Free-radical reactions and polycondensation of organic compounds are actually responsible for the synthesis of the organic matter (humic and fulvic acids) in soils (Wong, 2008). Based on these considerations, all microorganisms capable to produce the described battery of enzymes might find application in the depletion of recalcitrant compounds in environmental matrices, both by oxidizing the latter or catalyzing their polycondensation or humification (Ghosal et al., 2016).

Historically, white rot Basidiomycetes have been used in bioremediation processes (Baldrian, 2004; Schmidt et al., 2005). However, Ascomycetes have been found to be more adaptable to polluted environments, as they do not require co-metabolic carbon sources to trigger the secretion of the extracellular enzymes. Even though they are less often used for bioremediation applications, the interest in Ascomycetes is increasing, as a number of studies have demonstrated their ability to transform recalcitrant compounds (Covino et al., 2015; Becarelli et al., 2019; Siracusa et al., 2020), as well as their involvement in the synthesis of soil organic matter, and their ability to catalyze extracellular polymerization of polyphenols (Zavarzina et al., 2010).

In natural environments, bacteria may utilize two different approaches to exploit carbon sources: specialist species are able to utilize a unique carbon source, present at high concentrations (Kuenen, 1983), while generalist species are able to utilize a wide range of carbon sources, at low concentrations in the environment (Kuenen, 1983). In a polluted environment, it might be assumed that specialist species are expected to be responsible for the transformation of the contamination, since they can utilize the contaminant as a carbon source. However, the role of generalist species is also important because their metabolic flexibility enables them to establish their dominance in disturbed ecosystems and even support the ecological structure of communities in polluted environments (Jiao et al., 2016; Salem et al., 2019). However, their role in the bioremediation of pollutants in the environment is not yet evident.

In this study, a novel Ascomycetes was isolated from soil contaminated with TPH (8,538 mg/kg) collected at an abandoned oil refinery in Italy. The strain, taxonomically identified as a *Ciboria* sp., was able to utilize diesel oil as a sole carbon source. The *Ciboria* sp. strain was bioaugmented to the polluted soil that was co-composted with lignocellulose residues for the depletion of TPH. Bacterial 16S and fungal ITS metabarcoding was used to analyze microbial diversity in the soil during the process of TPH depletion. Moreover, functional metagenomic predictions for the bacterial community were inferred to envisage the role of the different bacterial taxa in the process of degradation of TPH. The concentrations of humic and fulvic acids in the soil were measured in parallel to the changes in TPH concentrations throughout the study. A correlation between the microbial diversity, TPH depletion, and the synthesis of the soil humic and fulvic acid fractions during the process of decontamination was established. The present study improves our understanding of the relationship between microbial biodiversity and bacterial and fungal interaction during the process of TPH depletion, providing perspectives in terms of optimization of the dedicated bio-based processes.

## Materials and Methods

### Polluted Soil, Woodchip, and Chemicals

The soil contaminated with TPH, at a concentration of 8,538 mg/kg, was collected at a decommissioned oil refinery in Trieste, Italy (45°36′16.9″N; 13°47′56.4″E). The diesel oil was purchased from a local service station. The woodchips used in the experiment were commercial lignocellulosic chips from chestnut (Cippato di castagno selezionato, KB, Italy). All other chemicals used in this study were of analytical grade and purchased from Merck (Milan, Italy).

### Isolation of the Fungal Strain

The fungal strain was isolated using selective pressure by incubating 1 g of soil in 100 ml of Basal Salt Medium (BSM: Na_2_HPO_4_, 2.2 g; KH_2_PO_4_, 0.8 g; NH_4_NO_3_, 3.0 g/L). The medium was enriched with 1% v/v of diesel oil (8,764 ± 63 mg diesel oil/ml) as sole carbon source. Gentamicin μg/L (500 μg/ml), chloramphenicol (50 μg/ml), tetracycline (125 μg/ml), streptomycin (50 μg/ml), and ampicillin (100 μg/m) were added to inhibit saprophytic bacterial growth. The flasks of 500 ml in volume were incubated for 2 weeks in a dark environment in orbital shaker at 120 rpm at 24 ± 1°C. After 15 days, a 10-ml aliquot was transferred to fresh BSM enriched with 1% v/v of diesel oil. The passage was repeated five times, then serial dilutions of the suspensions were plated on malt extract agar (MEA) plates (MEA: malt broth, 20 g; yeast extract, 5 g; agar, 15 g/L). A total of 1 ml of filtered (Whatman membrane filter paper Grade 1574 1/2) diesel oil was added on the top of the agar plates. The agar plates were incubated for 72 h in dark conditions at 24 ± 1°C. One fungal isolate was identified after visual inspection.

DNA of the fungal isolate was extracted using the FastPrep 24 homogenizer and FAST DNA Spin kit for soil (MP Biomedicals) according to the manufacturer’s protocol. Purity and quantity of DNA were assessed using the Qubit 3.0 Fluorometer (Thermo Fisher Scientific). The fungal strain was identified by amplification and sequence analysis of the 18S rDNA Internal Transcribed Spacer (ITS), using the ITS1 forward primer (5′-TCCGTAGGTGAACCTGCGG-3′) and ITS4 reverse primer (5′-TCCTCCGCTTATTGATATGC-3′) (White et al., 1990). The PCR products obtained were purified, sequenced on both strands, and aligned to sequence databases using BLASTN (Altschul et al., 1997). The morphological identification of the fungal isolate was performed by Mycoteca Universitatis Taurinansis (MUT).

### Fungal Degradation of Diesel Oil

The capacity of fungal strain to utilize diesel oil as a sole carbon source was verified in 100-ml flasks containing BSM amended with 1% v/v of diesel oil. A total of 12 flasks closed with glass stoppers were inoculated with 3 mg of fungal biomass derived from the maintenance of the strain in flask in malt extract broth (MEB). Before the inoculation of the fungal strain in BSM enriched with 1% of diesel oil, the liquid MEB fraction was removed from the fungal biomass by vacuum filtration on Whatman membrane filter paper Grade 1574½. The biomass was successively washed three times with sterile saline solution (0.9% NaCl), to remove residual MEB. Twelve (12) control flasks were not inoculated. All the flasks were incubated on an orbital shaker at 150 rpm at 28 ± 1°C, in the dark. Three flasks for time of analysis (0, 9, 13, 21 days) were extracted. At each sampling time, the flasks were separated in the supernatant and the fungal biomass by centrifugation at 2,000 × *g* at 4°C. The two portions were analyzed for diesel oil content. Biomasses and supernatant from each flask were extracted in CH_2_Cl_2_ and analyzed by (GC–MS) as described in Supplementary Materials. The fungal strain growth was quantified in terms of dry weight increment by sampling the entire fungal biomass of two separated flasks, vacuum filtering it with Whatman membrane filter paper Grade 1574½, drying at 60°C overnight, cooling in a desiccator, and weighting. The fungal biomass increase was calculated as follows: (fungal mass _*Ti*_ - fungal mass _*T0*_)/fungal mass _*T0*_)^∗^100, where T_0_ = initial time, T_*i*_ = each sampling time following the initial one (T_0_).

### Mesocosms Setup

A total of nine mesocosms (three replicates for each condition) were set up in glass pots containing 3 kg of TPH polluted soil (8,754 ± 650 mg/kg). Prior to incubation, the sediments were air dried and mixed with 10% (f.w./f.w.) of wood chips. The C/N/P ratio was adjusted at 100:10:1 by diluting 0.5 M solutions of NH_4_NO_3_ and KH_2_PO_4_ in water, adjusted to pH 7.3 by NaOH. The water content was adjusted at the 60% of the maximum water holding capacity. The pots were amended with the fungal inoculum as follows: control mesocosms (C) inoculated with 7% of autoclaved fungal biomass f.w./f.w. (fungal biomass/soil), F1 mesocosms inoculated with 1% of fungal biomass f.w./f.w. (fungal biomass/soil), F7 mesocosms inoculated with 7% of fungal biomass f.w./f.w. (fungal biomass/soil). The fungal biomass was previously grown in MEB, filtered on Whatman membrane filter paper Grade 1574½, and washed in sterile NaCl 0.9% (w/v) solution in water, before the mixing with the soil. All pots were maintained for 90 days at 24 ± 1°C in dark conditions and analyzed every 30 days. Water content was controlled by weighting the pots every 3 days of incubation. Sampling of soil for chemical analysis and metagenomic DNA extraction was performed by accurately mixing each pot by hands, producing one sample by two steps of quartering and random sampling. The sample was divided in the portion for the quantification of TPH, humic and fulvic acids, and metagenomic DNA for metabarcoding.

### Quantification of Total Petroleum Hydrocarbons, Humic Acid, and Fulvic Acid in Soil Samples

Quantification of TPH in soil samples was performed on aliquots of 20 g of soil per pot, following a modified ISO16703:2004 method for total hydrocarbons C_10_–C_40_. For further details about the methodology, see the Supplementary Materials. Quantification of humic and fulvic acids (HFA) in soil samples was performed on aliquots of 10 g of soil per pot, following the VIII.1 method in ordinary supplement n° 185 of “Gazzetta Ufficiale n. 248″21/10/1999. For further details about the methodology, see the Supplementary Materials. D’Agostino and Pearson omnibus normality test was adopted on residuals of TPH and HFA quantifications.

### Metabarcoding Analysis

Bacterial and fungal biodiversity was analyzed at days 0, 30, 60, and 90. Total DNA was extracted from 1 g of soil and purified using the FastDNA SPIN Kit for Soil (MP Biomedicals), following the manufacturer’s instructions. DNA quantity was measured using a Qubit 3.0 spectrofluorimeter (Thermo Fisher Scientific, United States) with high sensitivity (HS) assay, while quality assessment was performed by measuring 260/230 and 260/280 ratio with Spectro Star Nano UV–Vis spectrophotometer (BMG Labtech). A total of 288 ng of DNA was used for the production of paired-end libraries and for sequencing the V4–V5 hypervariable regions of the bacterial 16S rRNA gene by using as primers 515F forward primer (5′-GTGCCAGCMGCCG CGGTAA-3′) and 907R reverse primer (5′-CCGTCAATTCCTTTGAGTTT-3′). Fungi were identified by amplification and sequence analysis of the 18S rDNA internal spacer region using the ITS1-5F forward primer (5′-GGAAGTAAAAGTCGTAACAAGG-3′) and ITS2-2043R reverse primer (5′-GCTGCGTTCTTC ATCGATGC-3′). The sequencing was performed by Novogene (Novogene Company Limited Rm.19C, Lockhart Ctr., 301-307, Lockhart Rd. Wan Chai, Hong Kong). The libraries for Illumina sequencing were prepared by Novogene using NEBNext Ultra DNA Library Pre Kit, following manufacturer’s recommendations and index codes were added. The library quality was assessed on the Qubit@ 2.0 Fluorometer (Thermo Fisher Scientific) and Agilent Bioanalyzer 2100 system. The library was sequenced on an Illumina platform, and 250 bp paired-end reads were generated.

### Data Analysis

Paired-end reads were demultiplexed and trimmed by Cutadapt plugin for Qiime2. Forward and reverse reads were assembled, quality filtered, chimera filtered and assigned to Amplicon Sequence Variants (ASVs) following Qiime2 v.2019.7 standard pipeline. Amplicon Sequence Variants (ASVs) clustering was performed using DADA2 workflow implemented in Qiime2, with two classifiers trained on the V4–V5 hypervariable region extracted from Greengenes 13_8 99% 16S sequences database and 18S rDNA Internal Transcribed Spacer ITS sequences from UNITE v 8.0 99% database, respectively, for bacterial and fungal sequences. To allow comparison between different samples, ASV abundances per sample data were normalized by rarefaction to the sample with the least number of sequences. Subsequent analyses of α-diversity indexes (Chao1 Richness, estimator, Shannon index, Gini–Simpson Index and rarefaction curves of observed species) and β-diversity by principal coordinate analysis (PCoA) based on UniFrac distance, canonical correspondence analysis (CCA), and related statistical tests were performed on R 3.6.3 using Phyloseq, Vegan, and Pheatmap packages (versions 1.30.0, 2.5-6 and 1.0.12), respectively. Parametric statistics (two-way RM ANOVA and related *post-hoc* tests) on chemical data were performed by GraphPad Prism 6. The functional metagenomic prediction for the bacterial community was inferred using PICRUSt2 v. 2.1.4-b for unstratified metagenome contribution based on EC numbers, and by Nagpal et al. (2019)^1^ for stratified contribution of bacterial community to specific KEGG levels. Contributions with a Pathway Exclusion Cut-off (PEC) of 80% (i.e., only contributions that were constituted by 80% of genes/enzymes implied in pathways of interest, were taken into account) were filtered from the output data of iVikodak global mapper analysis, and were processed by R v. 3.6.3. Graphical output was produced by ggplot2 package v. 3.3.2 and Pheatmap v. 1.0.12. The sequences have been deposited at the Sequence Read Archive (SRA) database, National Center for Biotechnology Information (NCBI) with the projects ID numbers PRJNA688285 and PRJNA688293 for bacteria and PRJNA688315 for fungi.

## Results

### Isolation and Characterization of the Fungal Strain

One fungal strain native to the TPH polluted soil was isolated after cultural enrichments in the presence of diesel oil as sole carbon source. The strain was taxonomically identified by 18S rDNA Internal Transcribed Spacer ITS sequencing, as a species in the genus *Ciboria* (accession number KX866906.1) with a homology in ITS sequences of 100%. The phylogenetic tree is reported in Supplementary Figure 1. Morphological identification was carried out by the Mycoteca Taurinensis, University of Turin, Italy (MUT). Any morphological traits (e.g., sexual reproductive apparatus) were exploitable for a phenotypical characterization of the strain. The strain was deposited at the MUT as *Ciboria* sp. MUT 5852.

The fungal strain was found to be able to deplete 96% of the diesel oil after 9 days of incubation in mineral medium containing 10% (v/v) diesel oil as the sole carbon source, with a 280% increase in dry cell weight by the end of the incubation period.

### Depletion of Total Petroleum Hydrocarbons and Synthesis of Humic and Fulvic Acids at Mesocosm Scale

**FIGURE 1:**
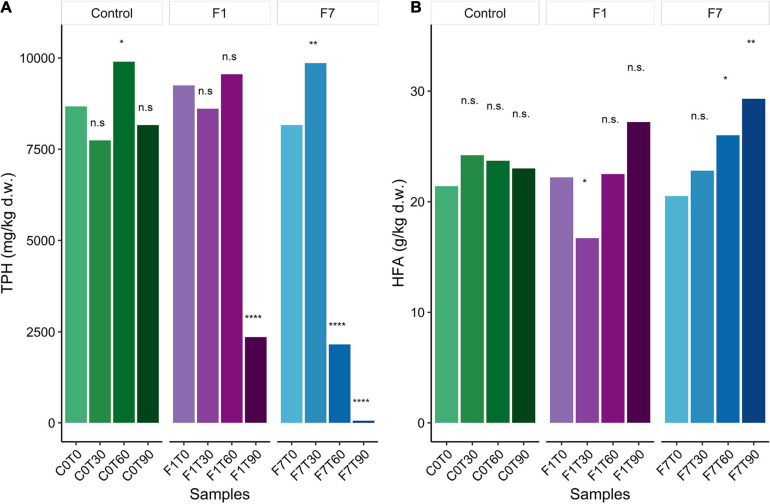
**(A)** Average of Total Petroleum Hydrocarbon (TPH) content for datapoint triplicates, expressed as mg/kg per dry weight (ppm); **(B)** Average of humic and fulvic acids (HFA) content for datapoint triplicates, expressed as g/kg per dry weight (‰). Statistical significance was assessed by two-way RM ANOVA test (Days = matching factor) followed by Dunnett *post-hoc* test corrected for multiple comparisons. Group labels represent statistical significance against 0 Days datapoint within each Treatment (C0, control mesocosms, F1, F7) at α = 0.05 : n.s., not significative, *corrected *p*-value < 0.05, **corrected *p*-value < 0.01, ****corrected *p*-value < 0.0001.

*Ciboria* sp. MUT 5852 was able to elicit and/or accelerate TPH transformation in the co-composting contaminated soil and higher rates of TPH degradation were observed in the F7 mesocosms inoculated with 7% f.w./f.w. (Figure 1A). More precisely, in F7 mesocosms, the 78% of TPH depletion was obtained after 60 days of incubation, and after 90 days of incubation, the depletion of TPH reached the 99%. In the F1 mesocosms (inoculated with 1% f.w./f.w.), 78% of TPH depletion was observed only after 90 days of incubation. In control mesocosms the TPH depletion was not significant over the 90 days of incubation, even though a trend to TPH depletion was observed (5.9%). Humic and fulvic acid (HFA) content in the co-composting soil was also measured every 30 days during the incubation (Figure 1B). In the control mesocosms, the concentrations of both humic and fulvic acids remained constant over the 90 days of incubation. Despite a general increase in the concentrations of humic and fulvic acids in all experimental conditions, a significant increase in their concentrations was observed only in the F7 mesocosms (31%). The significances for these quantifications are reported in Supplementary Tables 1–3 in Supplementary results.

### Bacterial Community Analysis

**FIGURE 2:**
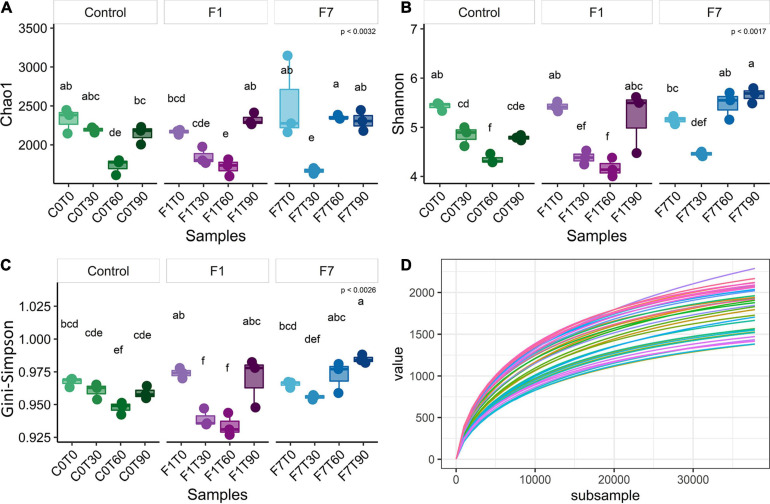
**(A)** Chao1 index, **(B)** Shannon index, **(C)** Gini–Simpson index (1-λ), and **(D)** rarefaction curves of observed species of bacterial community composition. Each sample was rarefied at 37,809 counts, and the distribution of three biological replicates per sample is reported. Box and whiskers hinges represent the first and third quartiles, upper and lower whiskers represent the furthest datapoints from median. Reported *p*-value is calculated by Kruskal–Wallis test (α = 0.05). Group letters represent statistical significance at α = 0.05, based on Fisher LSD *post-hoc* test, with Benjamini–Hochberg correction for multiple comparisons: groups sharing one or more letters are not significantly dissimilar.

The sequences passing the quality control were aligned and subjected to cluster analysis to ascertain their taxonomical affiliations. A total of filtered 2,062,918 reads were retrieved, more precisely 692,537 from control mesocosms, 686,101 from the F1 mesocosms and 684,280 from the F7. The α-Diversity was assessed based on the abundance of the various taxa within each sample triplicates, regarded as a community. In Figure 2, the rarefaction curves for observed species, the richness estimator (Chao1) and two diversity indices and are reported. These latter are the Shannon index, slightly biased toward common or more abundant species, and the Gini–Simpson index that is unbiased. Rarefaction curves showed that sequencing achieved a good coverage of biodiversity analyzed. The Chao1 estimator (Figure 2A) showed that changes in environmental conditions due to aeration, and the amendment of both bulking agent and macronutrients, affected the ASV richness in all the three experimental conditions, which showed similar trends, but with three different rates of variations. In control mesocosms, a significant decrease in richness is obtained after T60, with recovery within the following 30 days (T90). In F1 mesocosms, a slightly faster decrease was observed, with a following recovery at T90. In F7 mesocosms, a marked decrease was observed at T30, followed by a recovery at T60, anticipated with reference to the other experimental conditions. The Shannon index (Figure 2B) indicated a similar trend in all the experimental conditions. After an initial decrease, each experimental condition was successively associated with a recovery in the corresponding values. In control mesocosms, the decrease in species richness continued up to T60. By T90, the richness index increased to reach values close to those observed at T30. In F1 mesocosms at T30 and T60, the richness decreased, reaching similar values as the control condition at T60. At T90, the richness index increased to reach the values observed at the beginning of experiments (T0). In F7 mesocosms, the index decreased between T0 and T30. An increase in richness, when compared with the other experimental conditions, was observed at T90, and already at T60, the index reached the values of the T0. The Gini–Simpson index (Figure 2C) showed a similar trend; however, in the control and F7 mesocosms, the decrease in species evenness was less evident (i.e., C0T0–C0T30 and F7T0–F7T30 are not statistically different), suggesting that common or more abundant bacterial species were more affected than the rare or less abundant ones.

**FIGURE 3:**
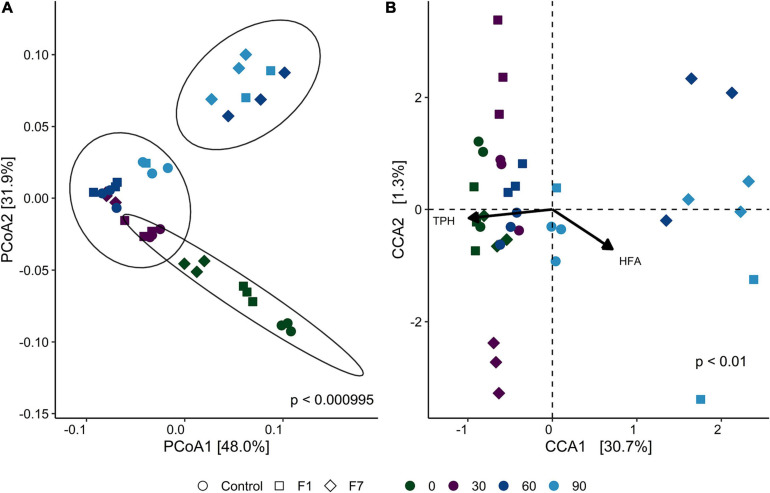
**(A)** Principal component analysis (PCoA) of bacterial communities, based on weighted UniFrac distance for each sample triplicate. Point shapes represent treatment category, while color indicates time category. The percentage reported on axes represent the amount of total variance depicted by each of them. *P*-value was calculated by ADONIS function (Vegan R package) between weighted UniFrac distances and sample groups, using the Bray–Curtis method with 1,000 repetitions. UPGMA hierarchical clustering was performed on the same distances. Circles enclose sample groups, which share a dendrogram height = 0.20. **(B)** Canonical correspondence analysis (CCA) biplot that shows correlation between amplicon sequence variant (ASV) composition of each sample triplicate and the environmental parameters total petroleum hydrocarbon concentration (TPH) and humic and fulvic acid concentration (HFA). Point shapes represent treatment category, while color indicate time category. Black arrows are the eigen-vectors representing constraining variables. Eigen-values of constrained axes are 0.16211 for CCA1 and 0.00856 for CCA2. Of the total inertia, 32% is explained by CCA1 and CCA2 axes. Reported *p*-value is calculated by PERMANOVA test performed on full model ASV − TPH + HFA with 999 permutations.

β-Diversity, as displayed by principal coordinate analysis (PCoA), provides an overview of the similarities in the bacterial communities between treatments with time of incubation. Figure 3A shows the PCoA based on weighted UniFrac distance between samples (each replicate is reported separately). Ellipses enclosed a 95% similarity in datapoints and showed three groups: the first group encloses all mesocosms at the beginning of experimentation (T0), with a partial overlying with the second ellipse. This ellipse encloses all mesocosms at 30 days (T30), plus control mesocosms at 60 (T60) and 90 (T90) days, and mesocosms with 1% fungal inoculum at 60 days (F1-T60). Last separate ellipse enclosed last timepoints for both inoculated mesocosms (F1-T90; F7-T90), and the treatment with 7% inoculum at 60 days (F7-T60). One of the samples of 1% inoculum at 90 days (F1-T90) was an outlier.

In CCA biplot (Figure 3B), the disposition of datapoints toward the unconstrained variables provide reported (TPH and HFA) evidence of two groups. The group on the right, corresponding to low concentrations of TPH, contained the F7 mesocosms at T60 and T90 and the F1 mesocosms at T90, suggesting a correlation between the bacterial diversity of the mesocosms and the process of TPH depletion. The same correlation was not observed for the control mesocosms. The correlation, recorded for F1 mesocosms at T90, was observed at T60 for F7 mesocosms. Statistically significant indexes for the bacterial communities are shown in Supplementary Tables 4, 5.

**FIGURE 4:**
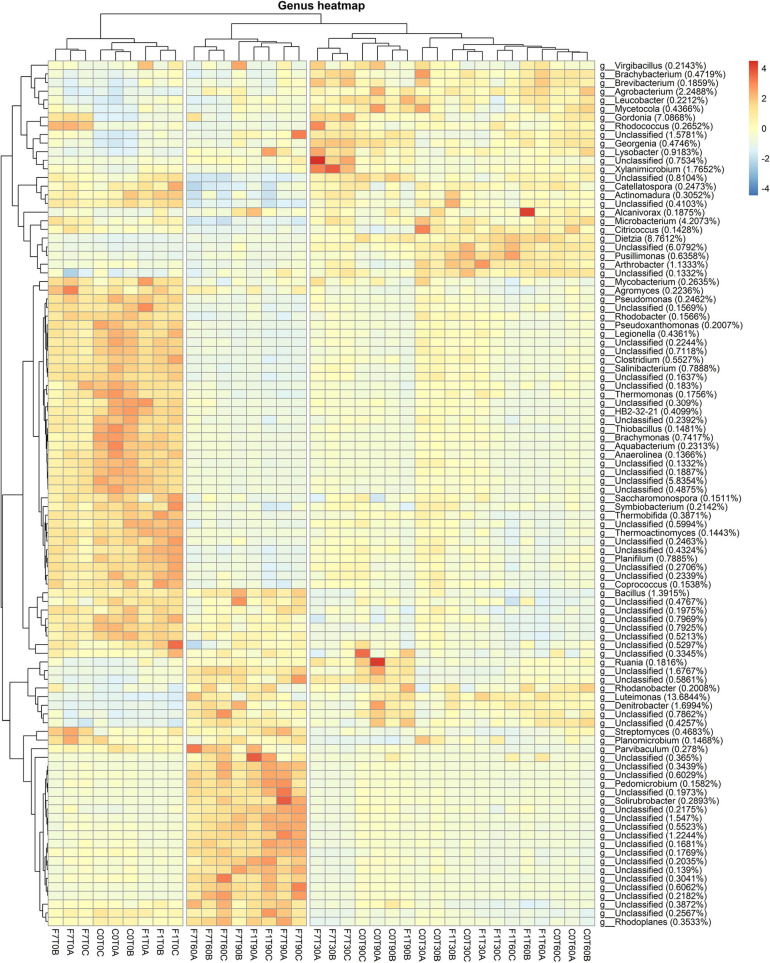
Heatmap showing bacterial ASV abundances per sample at genus level with a cut-off of 0.01%. Hierarchical clustering was performed on both rows and columns by Pearson correlation, based on Euclidean distance. Color scheme represents row-wise Z-scores of ASV counts. Percentage reported near ASV names represent the relative abundance of the sum of ASV counts per sample against total sum (i.e., Z = 0 matches reported percentage).

Results related to the taxonomic profiles of the bacterial communities across the mesocosms at genus level are reported in Figure 4. For a detailed description of taxa abundances at phylum and family level during the process of TPH depletion, see the Supplementary Materials and related figures: Supplementary Figures 2, 3. The hierarchical cluster analysis of all the mesocosms, based on their relative abundances and standard scores, divided the latter in three different groups. One group was composed of all the mesocosms at T0. A second group was composed of the mesocosms where TPH depletion occurred: the F7 mesocosms at T60 and T90, and the F1 mesocosms at T90. The third group was composed of all the mesocosms where, even though not statistically significant, a trend to TPH depletion with time of incubation was observed: the control mesocosms at the different times of incubation and all the inoculated mesocosms, with the exception of the F1 mesocosm at T90 and the F7 mesocosms at T60 and T90. All these mesocosms, preceding the onset of the TPH depletion, cluster in the same group, and defined as “transitional mesocosms.”

As a general consideration, higher average values for relative abundances for taxa are interpreted as an indication of an increase in representativeness of the corresponding taxa within the overall bacterial community. Higher values were evidently reached for some taxa in the mesocosms at T0 and in the mesocosms where the TPH depletion occurred, suggesting a higher representation of the corresponding taxa at the time of mesocosm set-up and at the time the TPH depletion was observed. Some of the latter were associated to similar percentages in both groups of mesocosms. These were especially unclassified bacteria, the *Bacillus* sp., belonging to *Firmicutes*, the *Ruania* sp. belonging to *Actinobacteria*. The *Ruania* sp. was recovered at higher percentage also in the control mesocosms at T90 and the F7 mesocosms at T30, both associated to a positive trend in initiating the process of TPH depletion. Some of the genera with high average frequency percentages recorded in mesocosms at T0 showed a decrease in frequency in the transitional mesocosms and a successive recovery in the mesocosms where TPH depletion occurred. These genera were the *Streptomyces* sp. of the Phylum *Actinobacteria*, *Rhodoplanes*, and *Parvibaculum* spp. of the Phylum *Proteobaceria*, and *Planomicrobium* spp. of the Phylum *Firmicutes*.

Considering relative abundances higher than 1%, the most abundant genera in the transitional mesocosms were *Agrobacterium*, *Gordonia*, *Xylanimicrobium*, *Microbacterium*, *Dietzia*, and *Arthrobacter* spp. The genera characterizing the transitional mesocosms were mainly in the Phylum *Actinobacteria*, apart from *Virgibacillus* sp. in the Phylum *Firmicutes* and *Agrobacterium* and *Lysobacter* spp. in the Phylum *Proteobacteria*. On the other hand, in mesocosms at T0 and where the TPH depletion occurred, the most abundant genera were in the Phylum *Proteobacteria*, even though, both groups of mesocosms were characterized by a diverse distribution of taxa among the different phyla, comprising *Proteobacteria*, *Actinobacteria*, *Firmicutes*, *Bacteroidetes*, and *Chloroflexi*. More precisely in the mesocosms at T0, the genera showing higher average percentages, decreasing in values during the process of TPH depletion, were *Pseudomonas*, *Legionella*, *Psuedoxhantomonas*, *Rhodobacter*, *Thermomonas*, *Brachymonas*, *Aquabacterium*, and *Thiobacillus* spp., in the Phylum *Proteobacteria*; *Mycobacterium*, *Agromyces*, *Saccharomonas*, *Thermobifida*, *Thermoactinomyces*, and *Salinibacterium* spp. in the Phylum *Actinobacteria*; *Clostridium*, *Coprococcus*, *Symbiobacterium*, and *Planifilium* spp. in the Phylum *Firmicutes*; and *Anaerolinea* spp. in the Phylum *Chloroflexi*. In the mesocosm where TPH depletion occurred, the genera gaining higher average percentages in frequency were *Rhodanobacter*, *Luteimonas*, and *Denitrobacter* spp. The genera associated with higher values in the transitional mesocosms were *Rhodoplanes* and *Pedomicrobium* spp. in the Phylum *Proteobacteria*, and *Streptomyces* and *Solirubrobacterium* spp. in the Phylum *Actinobacteria.* The genera *Rhodanobacter*, *Luteimonas*, and *Denitobacter* spp. were also associated with higher frequency values in the transitional mesocosm. *Luteimonas* spp., with an average value of 13.6%, was present in both transitional mesocosms and in mesocosms where TPH depletion occurred. It is worth noting that among the identified ASVs all across the samples, 52% were unclassified genera, and 30% of these were identified in mesocosms where TPH depletion occurred. All over the mesocosms, some of the unclassified ASVs reached significant percentages of the total population, with values up to 6% in the transitional mesocosms, 5% in the T0 mesocosms, and 1.6% in the mesocosms where TPH depletion occurred.

### Fungal Community Analysis

**FIGURE 5:**
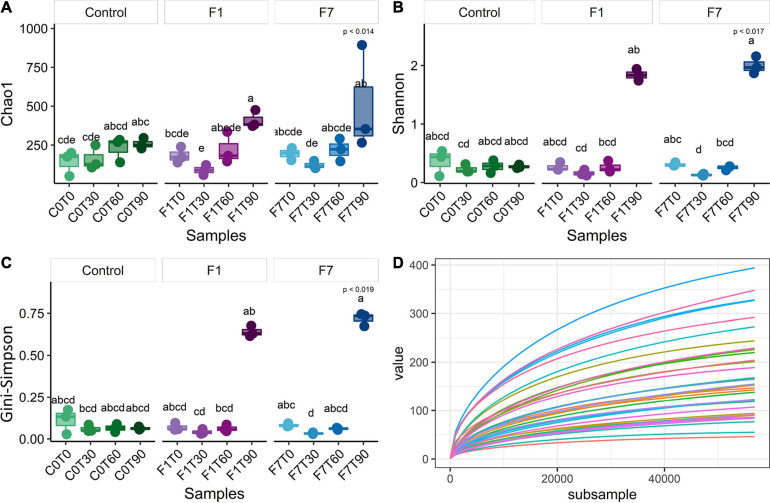
**(A)** Chao1 index, **(B)** Shannon index, **(C)** Gini–Simpson index (1-λ), and **(D)** rarefaction curves of observed species of fungal community composition. Each sample was rarefied at 56,830 counts, and the distribution of three biological replicates per sample is reported. Box and whiskers hinges represent first and third quartiles; upper and lower whiskers represent the furthest datapoints from median. Reported *p*-value is calculated by Kruskal–Wallis test (α = 0.05). Group letters represent statistical significance at α = 0.05, based on Fisher LSD *post-hoc* test, with Benjamini–Hochberg correction for multiple comparisons: groups sharing one or more letters are not significantly dissimilar.

A total of filtered 2,886,060 reads were retrieved from the 18S rDNA sequence analyses: 972,726 from control mesocosms, 944,401 from the F1 mesocosms, and 968,933 from the F7 mesocosms. Rarefaction curves showed a good coverage of diversity. The α-diversity of the fungal community was assessed, based on the number and abundance of various taxa within each sample and the Chao1 richness, Shannon index, Gini–Simpson index, and rarefaction curve based on observed species were determined (Figure 5). In terms of richness, a very slight increase in diversity with time of incubation was observed only in F1 mesocosms, even though a significant scatter of datapoints in F1 at T90 suggests a still evolving situation at least for rare or less abundant species. The other indexes did not show any significant variation during the time of incubation of all the other mesocosms.

**FIGURE 6:**
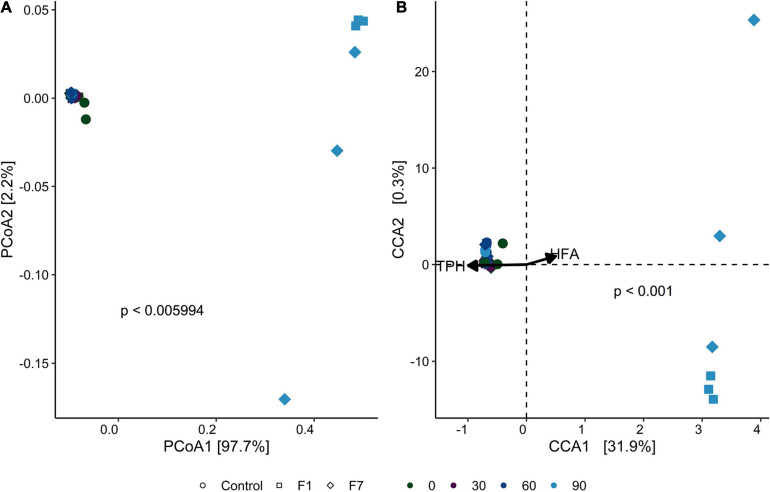
**(A)** Principal component analysis (PCoA) of fungal communities, based on weighted UniFrac distance for each sample triplicate. Point shapes represent treatment category, while color indicates time category. The percentage reported on axes represent the amount of total variance depicted by each of them. *P*-value was calculated by ADONIS function (Vegan R package) between weighted UniFrac distances and sample groups, using the Bray–Curtis method with 1,000 repetitions. **(B)** Canonical correspondence analysis (CCA) biplot that shows correlation between ASV composition of each sample triplicate and the environmental parameters total petroleum hydrocarbon concentration (TPH) and humic and fulvic acid concentration (HFA). Point shapes represent treatment category, while color indicates time category. Black arrows are the eigen-vectors representing constraining variables. Eigen-values of constrained axes are 0.3952 for CCA1 and 0.0042 for CCA2. Of the total inertia, 32% is explained by CCA1 and CCA2 axes. Reported *p*-value is calculated by PERMANOVA test performed on full model ASV − TPH + HFA with 999 permutations.

β-Diversity (Figure 6A) divided the mesocosms in two groups: the group on the right comprising the F1 and F7 mesocosms at T90, detached from the other cluster on the left, comprising all the other mesocosms. The CCA analysis (Figure 6B) showed that both F1 and F7 mesocosms at T90 correlated with low concentrations of TPH, suggesting an involvement of the fungal ecology of the two sets of mesocosms in the process of TPH depletion. Statistically significant indexes for the fungal communities are shown in Supplementary Tables 4, 5 in the Supplementary Materials.

**FIGURE 7:**
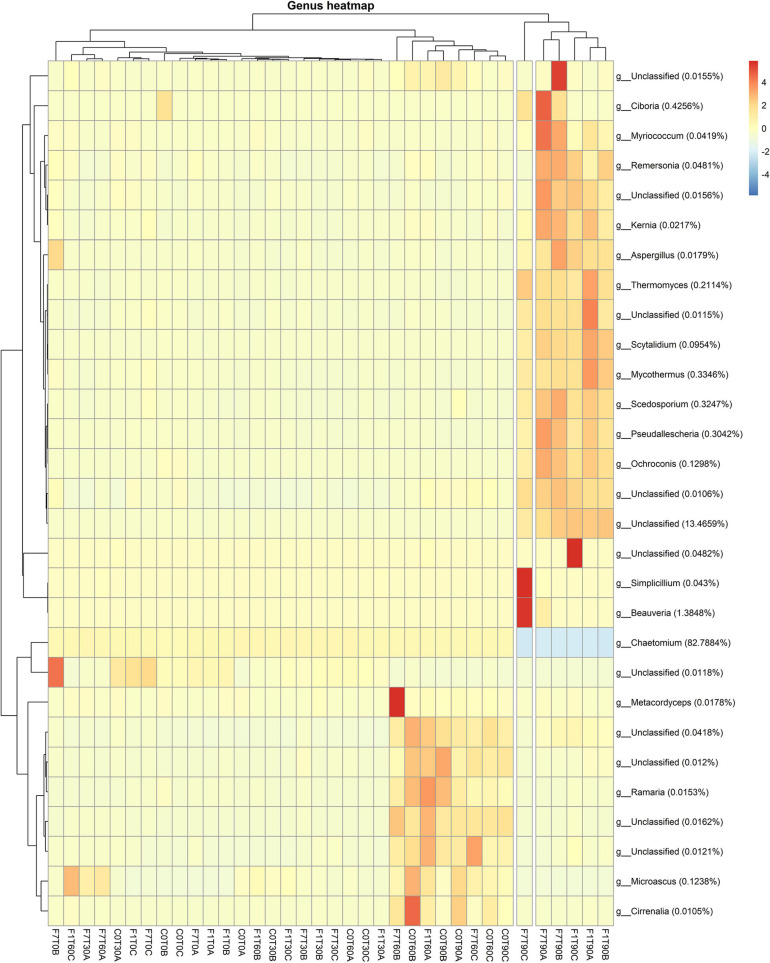
Heatmaps showing fungal ASV abundances per sample at genus level with a cut-off of 0.01%. Hierarchical clustering was performed on both rows and columns by Pearson correlation, based on Euclidean distance. Color scheme represents row-wise Z-scores of ASV counts per sample. Percentage reported near ASV names represents the relative abundance of the sum of ASV counts per sample against total sum (i.e., Z = 0 matches reported percentage). Unclassified groups were not pooled.

Taxa abundance at genus level during the whole process indicated that the mesocosms were divided mainly in two groups (Figure 7). The first group included the F1 and F7 mesocosm at T90; the rest of the mesocosms formed the second group. The unclassified fungi accounted for 13.5% as medium values, showing higher values in the F1 and F7 mesocosms at T90. *Cheatomium* sp. was the dominant genus with a medium value of 82.78% and at fairly constant level in all the mesocosms, showing a slight decrease in the F1 and F7 mesocosms at T90. Interestingly the *Ciboria* sp. reached higher level than the average in the F7 mesocosms at T90. Additional data on the taxa abundance at the phylum and family levels are reported in Supplementary Figures 4, 5.

### Bacterial Functional Metagenomic Prediction

**FIGURE 8:**
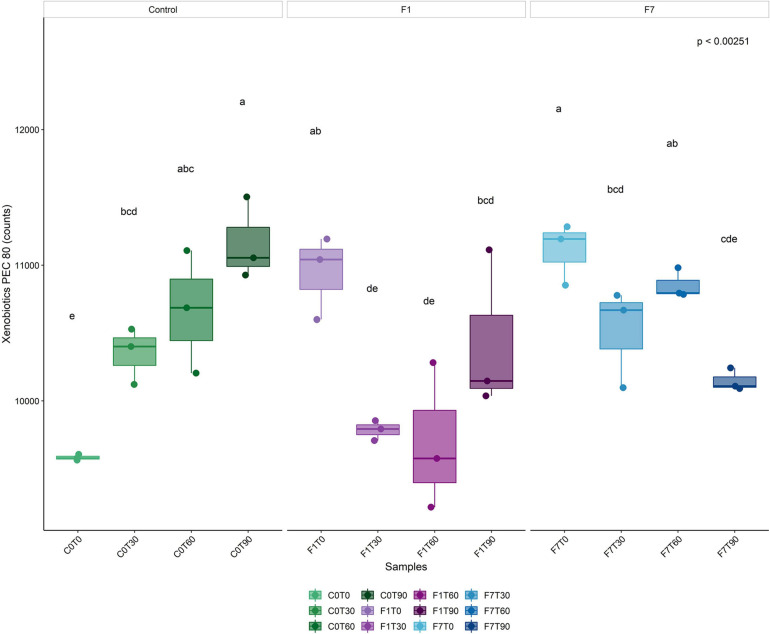
Boxplot representing total bacterial contributions to Xenobiotic biodegradation functional profile (Level 2) with a Pathway Exclusion Cut-off (PEC) of 80% (i.e., only contributions that contain 80% of genes/enzymes that constitute the pathway are taken into account), as calculated by iVikodak server. Box and whiskers hinges represent first and third quartiles; upper and lower whiskers represent the furthest datapoints from median. Reported *p*-value is calculated by Kruskal–Wallis test (α = 0.05). Group letters represent statistical significance at α = 0.05, based on Fisher LSD *post-hoc* test, with Benjamini–Hochberg correction for multiple comparisons: groups sharing one or more letters are not significantly dissimilar.

To better evaluate the metabolic potential of the different bacterial taxa during the process of TPH depletion, functional metagenomes of the bacterial community was predicted. In order to identify bacterial taxa potentially responsible for the depletion of TPH, the contribution of the different taxa to abundances of functional features of interest was analyzed. The increase in average percentages of contribution to the pathways was considered an indication of the putative involvement of the corresponding taxa to the functional features. Predicted proteins were classified by their Enzymatic Commission (EC) number, resulting in the identification of 2,313 ECs, and by KEGG orthology resulting in the identification of 7,374 KOs, across all samples. Metabolic reconstruction of pathways was carried out in iVikodak with a Pathway Exclusion Cut-off (PEC) of 80% (i.e., only contributions that contain 80% of genes/enzymes that constitute the pathway were retrieved). Results obtained showed that the Xenobiotic Biodegradation and Metabolism Module (KEGG Pathway 1.11) and related maps resulted to be among the top modules recovered across all samples (Figure 8). In both the inoculated mesocosms, F1 and F7, a significant increment in the corresponding counts at the time of the inoculation of the *Ciboria* sp. was recorded, followed by a decrease to lower levels at T30. In F1 mesocosms the reached values were maintained up to T60. Successively, an increase in the corresponding values was observed at levels lower than the one at T0. In F7 mesocosms a slighter decrease in counts was observed at T30. The values were maintained up to T60. A further decrease at values lower than the one at T0 occurred at T90.

**FIGURE 9:**
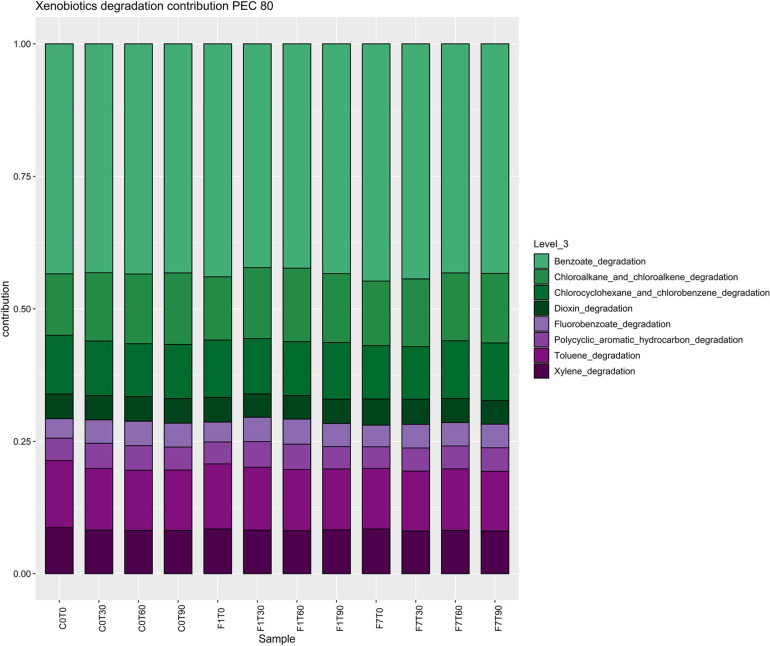
Stacked barplot representing composition of bacterial contributions to xenobiotic degradation pathways, depicted in Figure 8. Each bar represents the average of three replicates.

**FIGURE 10:**
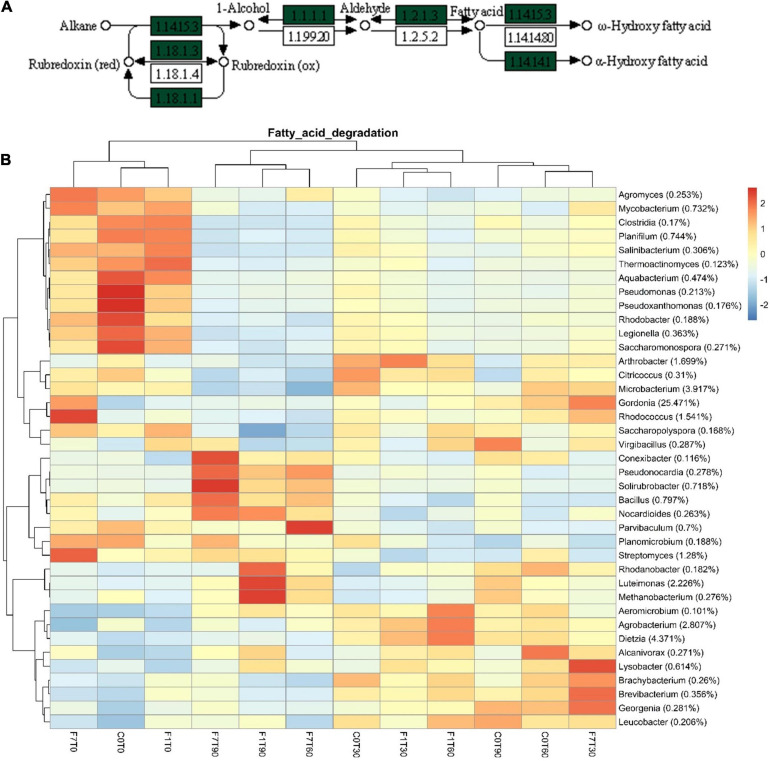
**(A)** Particular of fatty acid degradation pathway map (map00071) depicting aliphatic hydrocarbon degradation. Green labels indicate the presence of enzymatic features among those inferred in metagenome. **(B)** Heatmap showing bacterial contributions to fatty acid degradation pathway in metagenome, as calculated by iVikodak server, with a PEC of 80%. Reported taxonomic resolution is genus level, with a cut-off of 0.1%. Hierarchical clustering was performed on both rows and columns by Pearson correlation, based on Euclidean distance. Color scheme represents row-wise Z-scores of contribution counts per ASV. Percentage reported near ASV names represents the relative abundance of the sum of ASV contributions per sample against total sum (i.e., Z = 0 matches reported percentage).

In the Xenobiotic Biodegradation and Metabolism Module, different KEGG maps of interest were recovered all over the mesocosms, indicating the potential presence of different pathways capable of transforming the aromatic fraction of the contamination such as benzoate (map00362), toluene (map00623), xylene (map00622), and even halogenated compounds, fluorobenzoate (map00364), chloroalkane and chloroalkene, map00625, chlorocyclohexane, and chlorobenzene (map00361) (Figure 9). The fatty acid degradation pathway harboring the alkane monooxygenase (map 00071) for the oxidation of the saturated fraction of TPH was also present (Figure 10).

The contribution of the single bacterial genera to the predicted functional features is shown in Supplementary Figures 6–13. The functional feature distribution in the different mesocosms was clustered in the same three groups: the mesocosms at T0, the transitional mesocosms, and the mesocosms where the TPH depletion occurred. More precisely, in the mesocosms at T0 all over the pathways, the genera that showed the higher contribution were *Agromyces*, *Mycobacterium*, *Saccharomonospora*, *Salinibacterium*, *Thermoactinomycetes*, *Saccharopolyspora*, *Gordonia*, *Rhodococcus*, *Citrococcus*, *Microbactrium*, and *Streptomyces* spp. in the Phylum *Actinobacteria*; *Rhodobacter*, *Acquabacterium*, *Pseudomonas*, *Pseudoxanthomonas*, *Thermomonas*, *Legionella*, and *Parvibaculum* spp. in the Phylum *Proteobacteria*; *Planimicrobium*, *Virgibacillus*, *Planifilium*, and *Bacillus* spp. in the Phylum *Firmicutes*. Actually, the genus *Thermomonas* sp. showed higher contribution only to the benzoate degradation pathway, while the genus *Thermoactinomycetes* showed a higher contribution in benzoate and in halogenated compound degradation pathways (Supplementary Figures 6–8, 10).

As a general trend all these taxa, contributing in mesocosms at T0, showed a decrease in their contribution in the transitional mesocosms, where different genera from the previously mentioned showed a significant increase in contribution. More precisely these latter were *Arthrobacter*, *Dietzia*, *Brachybacterium*, *Brevibacterium*, *Gordonia*, and *Leucobacter* spp. in the Phylum *Actinobacteria*; *Alcanivorax*, *Lysobacter*, and *Agrobacterium* spp. in the Phylum *Proteobacteria*. The mesocosms where TPH depletion occurred, were characterized by the higher contribution of some genera contributing also at T0, such as *Planomicrobium* and *Bacillus* sp. in the Phylum *Firmicutes*; *Streptomyces* sp. in the Phylum *Actinobacteria* and *Parvibaculum* sp. in the Phylum *Proteobacteria*. The contribution of these taxa was fairly low in transitional mesocosms. At the same time the contribution of the genera *Rhodanobacter*, *Luteimonas* spp. for *Proteobacteria*, *Nocardioides*, *Psuedonocardia*, *Solirubrobacter* spp. for *Actinobacteria* increased significantly in the mesocosms where TPH depletion occurred. These latter showed a significant lower contribution in transitional mesocosms and in mesocosms at T0. *Proteobacteria* showed a decrease in contribution of most of the taxa contributing at T0. Only *Parvibaculum* sp. restored its contribution in the mesocosms where TPH occurred. In the same mesocosms, *Rhodanobacter* and *Luteimonas* spp. were characterized by an increase in contribution, suggesting their involvement with *Parvibaculum* sp. in the depletion of TPH. The same trend was observed for genera in the Phylum *Firmicutes* that showed a recovery in the abundance of *Planimicrobium* and *Bacillus* spp., in mesocosms where TPH depletion occurred, suggesting that these genera were involved in TPH depletion. In relation to *Actinobacteria*, a decrease in biodiversity of the contributing taxa in the transitional mesocosms was observed, but in the transitional mesocosms, genera in the Phylum, *Actinobacteria* were dominant over the other Phyla. A recovery in contribution in the mesocosms where TPH depletion occurred was observed for *Streptomyces* sp. In the same mesocosms, *Nocardioides*, *Pseudonocardia*, and *Solirubrobacter* spp. were characterized by an increase in contribution, suggesting their involvement, with *Streptomyces* sp. in the depletion of TPH.

Apart from *Nocardioides* and *Pseudonocardia* that showed an average relative abundance lower than the 0.1%, all over the mesocosms, and *Luteimonas* that showed the 13.7% in average relative abundance, the other taxa showed average values among 0.14 and 1.39%.

**FIGURE 11:**
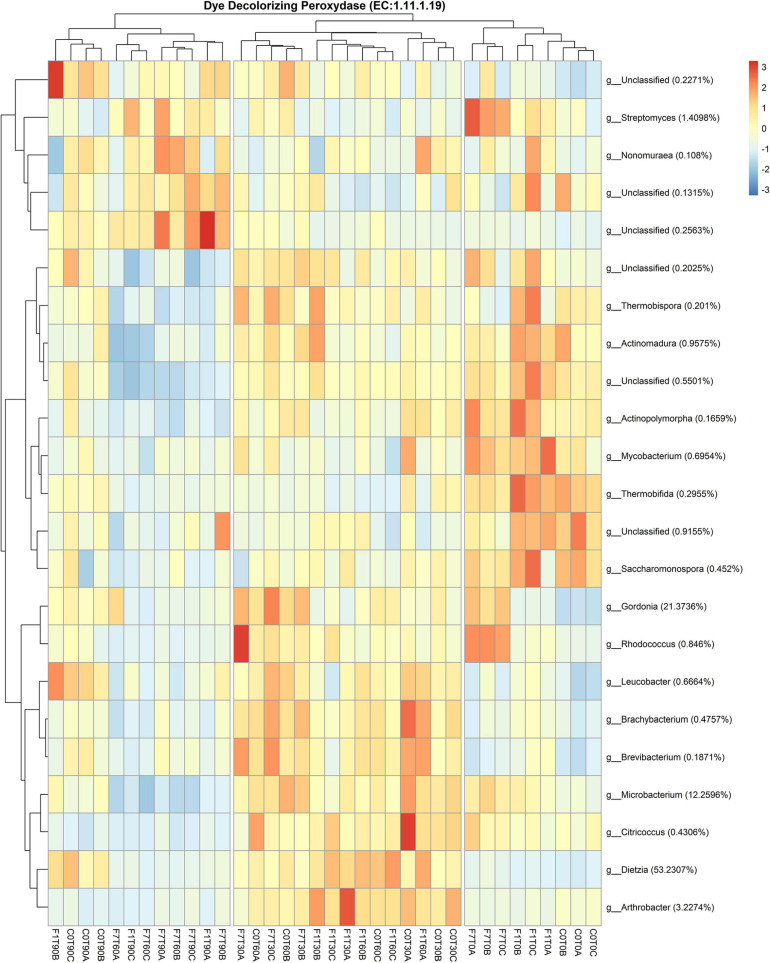
Heatmap showing Bacterial contribution to Dye decolorizing peroxidase (EC:1.11.1.19) in metagenome, as calculated by PICRUSt 2. Reported taxonomic resolution is genus level, with a cut-off of 0.1%. Hierarchical clustering was performed on both rows and columns by Pearson correlation, based on Euclidean distance. Colour scheme represents row-wise Z-scores of contribution counts per ASV. Percentage reported near ASV names represent the relative abundance of the sum of ASV contributions per sample against total sum (i.e. Z = 0 matches reported percentage). Unclassified ASV contribution were not pooled.

**FIGURE 12:**
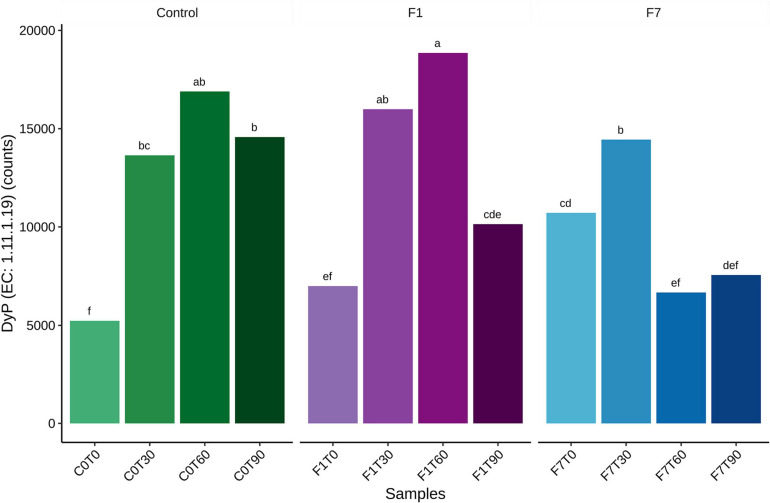
Average of total bacterial contributions to dye decolorizing peroxidase (DyP) for each datapoint triplicate. Statistical significance was assessed by Friedman test followed by Fisher LSD *post-hoc* test with Benjamini–Hochberg FDR corrected for multiple comparisons. Group labels represent statistical significance at α = 0.05. Groups sharing one or more letters are not significantly dissimilar.

A further analysis of the functional features of the bacterial community, revealed bacterial taxa encoding the Dye decolorizing peroxidases (DyP) (EC:1.11.1.19). The contribution of all the taxa to this functional feature in the three experimental conditions is reported in Figure 11. A total of 17 genera encoded the DyP genes. The highest contributions were due to *Dietzia* (53.23%), *Gordonia* (21.37%), *Microbacterium* (12.26%), *Arthrobacter* (3.23%), and *Streptomyces* (1.41%) spp., all contributing mostly to the transitional mesocosms, with the exception of *Streptomyces* sp., contributing mostly to the mesocosms at T0 and where TPH depletion occurred. In Figure 12, the profile of the DyP counts in all the experimental conditions was reported. In Table 1 the correlation between counts of the DyP, TPH, and HFA concentrations are reported. The correlations between the variables evidenced a progressive increase for TPH/DyP and TPH/HFA with the increase in fungal inoculum density. In fact, the reported correlation coefficients become very robust and positive for DyP/TPH couples, very robust and negative for TPH/HFA and not robust and negative for DyP/HFA. The observation that the profile counts of DyP (Figure 12) were similar in all the three experimental conditions, suggested that the strong correlation between DyP and the TPH content, limited to F7 mesocosms, was due to a covariation more than to a causal relation.

**TABLE 1:** Spearman’s rank correlation coefficient for combination of DyP total counts per sample and relative TPH and HFA concentrations, separated per mesocosm thesis.

ρ	TPH/EC:1.11.1.19	HFA/EC:1.11.1.19	TPH/HFA
Control	0.18	0.10	–0.04
F1	0.32	–0.40	–0.53
F7	0.88	–0.52	–0.82

*DyP, dye decolorizing peroxidases; TPH, total petroleum hydrocarbons; EC, enzymatic commission number; HFA, humic and fulvic acids.*

## Discussion

Fungi have been described as important in the degradation of TPH in polluted soils and sediments. In the last decade, fungi have been used in the design of bio-based processes for soil and sediment decontamination, both at lab and pilot-scale (Covino et al., 2016; Di Gregorio et al., 2016; Becarelli et al., 2019). The use of selected fungal strains, to elicit or optimize processes of biodegradation, might be important to transfer the promising fungal metabolism on the industrial scale. However, any intervention, when transferred on the real scale, must consider the impact of the inherent costs on the process. The aim of this work was the study and the optimization of the mycoaugmentation approach to the process of TPH depletion in historically polluted soil. The optimization was mainly based on the isolation of fungal strains autochthonous to the soil to be treated, efficient in the utilization of the contamination as carbon source, and the modulation of the density of the inoculum to be bioaugmented. The biodegradation of recalcitrant compounds like TPH in a complex matrix like soil is actually due to the combination of fungal and bacterial metabolism. The present work was focused on the study of both fungal and bacterial diversity during the process of TPH depletion, to highlight the synergism between the two communities.

The isolation and selection of a fungal candidate for mycoaugmentation was based on its capacity to use diesel oil as sole carbon source, and the isolated fungal strain was taxonomically characterized as a *Ciboria* sp. *Ciboria* sp. is actually quite rare in the environment. *Ciboria shiraiana* and *Ciboria caruncoloides* were retrieved as plant pathogens, causing sclerotiniose on *Morus alba* (Zhu et al., 2020). Their plant pathogenesis action relies on the production of cell wall degrading enzymes and secondary metabolites that acts as toxins to promote decomposition of plant cuticle (Blanc et al., 2018). However, a recent research by Perkins et al. (2019), related to the characterization of the fungal community of diverse groundwater boreholes in North-Eastern Germany, led to the isolation of a *Ciboria* sp. strain capable to transform polymeric Azo-dyes and aryl amines.

In this study, the effects of the *Ciboria* sp. inoculum density adjustment on TPH depletion kinetics in soil showed that the highest density was determinant to accelerate the TPH degradation process. In order to investigate the reason at the base of the observation, a molecular approach has been adopted. The results obtained were correlated to the two main parameters quantified during the process of decontamination, TPH depletion and the synthesis of humic and fulvic acids (HFA) in the soil in treatment. The two parameters were co-monitored because of evidence from other studies that showed that composting of TPH in soil can result in the transformation of polyaromatic hydrocarbon compounds into HFA compounds (Wong, 2008; Zavarzina et al., 2010). Actually, saprophytic fungi are described as responsible for the composting of the organic matter in soil (Ingold et al., 1993). However, in our work, a statistical correlation between the fungal ecology, its evolution, and the HFA increment was not observed. On the other hand, a correlation between TPH degradation and an increase in the ASVs for *Ciboria* sp. was observed in the F7 mesocosm at T90, where TPH depletion occurred. The ability of *Ciboria* sp. to utilize diesel oil as a sole carbon source suggests that the fungal strain is responsible for TPH depletion in the soil in treatment.

However, during the process of TPH depletion, any strong evidence of the capacity of the fungal inoculum to adapt to the soil conditions and to be dominant was recovered. Unfortunately, it should be noticed that the molecular approach here adopted to study the fungal ecology was affected by the amendment of the wood chip as bulking agent. In fact, the latter resulted to be colonized by the *Chetomiaceae* family (data not shown) that determined a tremendous increase of the taxon, in all the experimental conditions. The evaluation of the role of the entire fungal community might have been affected by the massive presence of the *Chaetomium* sp. In fact, a possible preferential amplification of the *Chaetomium* sp. ITS sequences might have biased NGS data, masking the rest of the under-represented fungal community. The rarefaction curve referred to the analysis of the fungal community suggested that after a relatively low number of sequencing reactions a plateau is rapidly reached, especially in the mesocosms where the bulking agent was amended. The effect is more evident with the increase of the time of incubation. Considering the potential richness in fungal biodiversity in a soil, the result here obtained on the fungal ecology might be an artifact and the adopted survey system, probably not sufficiently precise to properly monitor the bioaugmented fungal inoculum.

In this context, the main information gained, is related to the evidence that, even if dominant, as the case of the *Chaetomium* sp., an allochthonous fungal saprophyte was not directly responsible for the depletion of the contamination and humification of the organic matter. The result might be considered surprising, since the involvement of the extracellular enzymatic activity of saprophytic fungi has actually been extensively described as involved in the process of depletion of recalcitrant molecules, even though the fungal candidate was allochthonous to the matrix to be treated (Bardi et al., 2017; Siracusa et al., 2020; Spennati et al., 2020). However, the assumption might confirm the need to isolate microbial specimen autochthonous to environmental matrices to be treated, in order to exploit their competitive advantages on the complex microbial community.

In many cases of mycoaugmentation the goal of the decontamination has been achieved, but a detailed description of the interactions between the fungal inoculum and the autochthonous bacterial community in the matrix in treatment is missing. The co-occurrence of fungal and bacterial metabolism in the mycoremediation process has been suggested (Banerjee et al., 2016; Becarelli et al., 2019; Spennati et al., 2020). Confirming the hypothesis, our data suggest that not only the bacterial metabolism is pivotal to TPH depletion, but the addition of the fungal inoculum accelerated the TPH degradation process.

The bacterial functional features associated to the depletion of TPH were putatively ascribable to the capability of the bacterial community to transform both the saturated and the aromatic fraction of the TPH contamination. The occurrence of several bacterial metabolic modules, comprised in the xenobiotic metabolism and biodegradation pathways, such as the polycyclic aromatic hydrocarbons, fluorobenzoate, toluene, xylene, benzoate, and dioxin degradation pathways, suggests a specialization of the autochthonous bacterial community in metabolizing organic compounds containing aromatic moieties. In addition, the occurrence of bacterial alkane monooxygenases, in the module for the fatty acid degradation, suggests specialization of the autochthonous bacterial community in metabolizing the saturated fraction of TPH contamination. These functional features were associated with most of the bacterial taxa identified in the mesocosm treatments with relative percentages over the 0.1%. However, in this context, it is reasonable to limit the discussion to the physiological role of the bacterial taxa correlating to the process of TPH depletion in terms of both variations of relative abundances and frequencies of functional contribution to the process.

The diversified microbial community correlated with TPH depletion in the mesocosms at T0, decreased in diversity in the transitional mesocosms that showed a functional predominance of *Actinobacteria*. These latter, belonging to *Arthrobacter*, *Dietzia*, *Brachybacerium*, *Brevibacterium*, *Gordonia*, and *Leucobacter* spp. harbor also the dye-decolorizing peroxidases (DyP). These enzymes can be retrieved both in eukaryotic and prokaryotic organisms, showing a higher diversity in prokaryotes (Chen and Li, 2016). Bacterial DyP from class A to C have high redox potentials that allow both the oxidation of phenolic (Chen et al., 2015) and non-phenolic lignin model compounds (Min et al., 2015). These features could be involved in TPH transformation. However, any robust correlation between DyP counts and TPH depletion was observed here, as well as any correlation was observed with HFA synthesis. These evidences suggest that the DyP is associated to the saprophytic metabolisms of *Actinobacteria*, more than to their capacity to deplete the contamination.

The above mentioned saprophytic actinobacterial species characterized mainly the transitional mesocosms and lost their dominance in mesocosms where TPH depletion occurred, where an increment of predominantly actinobacterial hydrocarbonoclastic species was observed. These latter, the *Nocardioides*, *Pseudonocardia*, *Solirubrobacter*, and *Streptomyces* spp., apart from *Streptomyces* sp., were not characterized by the DyP, confirming that the DyP is associated with the saprophytic metabolisms of *Actinobacteria*, more than to their capacity to deplete the contamination.

Interestingly, some of the hydrocarbonoclastic actinobacterial species showed an initial relative abundance lower that 0.1%, as the case of *Nocardioides* and *Pseudonocardia* spp. The involvement of initially rare actinobacterial species in the depletion of the contamination was unexpected but emphasizes the importance of a detailed analysis of the microbial ecology during the process in study.

All in all, results obtained, described an initial competitiveness of actinobacterial saprophytic generalist species that preceded the establishment of specialist species. The inoculum of *Ciboria* sp. accelerated the shift from generalist to specialist *Actinobacteria* on a density weight ratio. This assumption suggests that the increase in the fungal inoculum is pivotal to construct a metabolic network between bacteria and fungi that end up with the acceleration of the process of decontamination. *Ciboria* sp. was not only responsible for the TPH depletion but also for harmonizing the interaction between the fungal and the bacterial hydrocarbonoclastic metabolism. The exact mechanisms involved merits further research, but results here obtained goes tentatively in the direction of a partial description. *Actinobacteria* are described for their fundamental role in nutrient cycles in the environment, responsible for the cycling of carbon, nitrogen, phosphorous, potassium, and microelements, replenishing the supply of nutrients in soil (Hill et al., 2011). As saprophytes, they produce a vast range of extracellular enzymes for the degradation of any carbon sources present in the environment, even if recalcitrant to biodegradation and present at limiting concentrations for growth. These metabolic capacities are involved in a general mobilization of carbon, with the consequent blooming of microbial commensal species, that favors the competitiveness of *Actinobacteria* also in oligotrophic environments (Eisenlord and Zak, 2010; Zhang et al., 2019). Somehow, the bioavailability of the contamination, especially in historically polluted sites, might render the polluted soil an oligotrophic environment, due to the lack of bioavailability of the dominant carbon source. The saprophytic metabolisms of fungi and *Actinobacteria*, if they have developed a resistance to the contamination, might be capable to trigger transformation process of the organic matter, determining a mobilization of the contamination. These capabilities, in a polluted soil, might be pivotal to the blooming of specialist species, capable to utilize preferentially or exclusively the contamination as carbon source. Thus, saprophytic generalists might result to be pivotal to prime the actual degradation of contaminants. In this context, the saprophytic metabolisms of the *Ciboria* sp. reinforced the effect, accelerating the onset of the process of TPH degradation.

The ecological traits already described in literature for the actinobacterial species, here involved in TPH depletion, confirm their envisaged role in the process. In relation to the generalist species, all of them were described as both capable to participate to the transformation of recalcitrant compounds and to the nutrient cycles in the ecological niches they colonize. *Arthrobacter* sp. has been described as capable of transformation of aromatic compounds, organochlorine, and pesticides (Gkorezis et al., 2015), and also described to play an important role in nitrogen cycle in soil (Cobo-Díaz et al., 2015). *Dietzia* sp. has been described for alkane and aromatic hydrocarbon degradation activities (Nazina et al., 2015). However, described as a bacterial species characterizing many environmental matrices, suggesting interesting adaptation capabilities, *Dietzia* sp., is still poorly described, and a new fundamental ecological role will be clarified soon. *Brachybacterium* sp. is involved in nitrogen cycle (Bai et al., 2019) and in hydrocarbon degradation (Wang et al., 2014). *Brevibacterium* sp., described as involved in crude oil transformation (Farahat and El-Gendy, 2008), is described also as involved in the nitrogen cycle (Baskaran et al., 2020). *Gordonia* sp., has been described as capable to transform diverse substrates comprising hydrocarbons and rubber (Drzyzga, 2012), but it is also involved in the cycle of phosphorous in soil (Hoberg et al., 2005). *Leucobacter* sp. has been described as capable to degrade hydrocarbons in polluted soil (Brzeszcz et al., 2020) and reported for its ligninolytic activity (Rahman et al., 2013), moreover involved in nitrogen cycle in soil (Solanki et al., 2017).

On the other hand, with reference to actinobacterial specialist species, *Solirubrobacter* sp. has been described as capable to deplete polycyclic aromatic hydrocarbons in polluted soils (Lu et al., 2019). *Streptomyces* sp., which is one of the most abundant *Actinobacteria* in the environment, accounting for almost 5% of the ∼16,000 described bacterial species, has been described as capable of transforming the recalcitrant lignin (Lewin et al., 2016) and petroleum hydrocarbons (Baoune et al., 2018). *Nocardoides* sp. has been described as involved in phenanthrene degradation (Saito et al., 1999). *Pseudonocardia* sp. has been described as involved in biodegradation of pyrene (Wang et al., 2016). Thus, also the key metabolic traits envisaged for the actinobacterial specialist species here involved in TPH depletion, are confirmed in literature.

The increase in bacterial biodiversity observed in inoculated mesocosms after 90 days of incubation, despite the decrease observed for *Actinobacteria*, was due to the contribution of specialist species belonging to *Proteobacteria* and *Firmicutes*. At T0 *Proteobacteria* were distributed among different genera but most of them lost their predominance in transitional mesocosms, where TPH depletion was triggered. This trend was not observed for *Luteimonas* and *Rhodanobacter* spp. that showed high values all over the mesocosms. *Proteobacteria* in transitional mesocosms showed an increase in contribution of *Agrobacterium* and *Lysobacter* spp. In mesocosms where TPH depletion occurred, the increase in contribution was associated to *Parvibaculum*, *Rhodanobacter*, and *Luteiomonas* spp. All in all, results showed that also *Proteobacteria* were subjected to a process of selection of different genera during the process of TPH depletion, with significant differences between mesocosms where the TPH depletion was triggered and mesocosms where the latter occurred. Moreover, also in the case of *Proteobacteria*, the inoculum of *Ciboria* sp. accelerated the shift from species characterizing the transitional mesocosms and the one characterizing the mesocosms where the TPH depletion occurred. In fact, the predominance of *Parvibaculum*, *Rhodanobacter*, and *Luteimonas* spp., competent for TPH depletion, was already evident at T60 lasting up to T90 in F7 mesocosms. The predominance of the same genera occurred only at T90 in F1 mesocosms and did not occur at all in the control mesocosms. Any of the aforementioned *Proteobaceteria* harbored the DyP, suggesting intracellular metabolisms of the specialists *Parvibaculum*, *Rhodanobacter*, and *Luteimonas* spp., for the utilization of the contamination as sole carbon source. *Parvibaculum* sp. is known to degrade both aromatic and aliphatic hydrocarbons (Shin et al., 2019). Both *Luteimonas* and *Rhodanobacter* spp. have been described as capable to degrade hydrocarbons (Gutierrez, 2019). *Rhodanobacter* sp. was described as capable to degrade of benzo(a)pyrene, biphenyl, naphthalene and lindane (Uhlik et al., 2012). Jones et al. (2014) described *Luteimonas* sp. as responsible for the co-metabolic transformation of benzo[a]pyrene. The genus was described also as a candidate of bacterial consortia capable to deplete pyrene in sediments (Bacosa and Inoue, 2015). *Luteimonas* sp. capability to degrade alkanes was also reported (Zheng et al., 2018).

With reference to *Lysobacter* and *Agrobacterium* spp. contributing to the phase of triggering of TPH depletion in the transition mesocosms, the two species are described in literature as associated predominantly to saprophytic lifestyle, producing extracellular enzymes such as cellulolytic enzymes (Palumbo et al., 2005), laccases (Ventorino et al., 2015), and both intracellular and extracellular alkane hydrolase (Sharma et al., 2019).

All in all, results indicate that also for *Proteobacteria* is possible to distinguish between putative generalist and specialist species that participate to the triggering and the effective phase of TPH depletion, respectively. The onset of the specialists over the generalists was also accelerated by the inoculum of *Ciboria* sp., confirming that the fungal inoculum is pivotal to construct a metabolic network between bacteria and fungi that end up with the acceleration of the process of decontamination.

On the other hand, in relation to *Firmicutes*, their participation to the TPH depletion was restricted to putative specialist species. The genera showing taxonomical and functional predominance at T0 were *Planomicrobium* and *Bacillus* spp. They decreased in representativeness in the transitional mesocosms to recover it in the mesocosms where the TPH depletion occurred, suggesting that *Firmicutes* are not participating to the triggering of the process of TPH degradation but, on the other hand, they are directly involved in their degradation, as specialists. Also, in the case of *Firmicutes*, the inoculum of *Ciboria* sp. accelerated the onset of specialist species during the process of TPH depletion. In fact, *Planomicrobium* and *Bacillus* spp. were predominant in F7 mesocosms already at T60, in F1 mesocosms later at T90 and they were never predominant in transitional mesocosms. Also. the *Firmicutes* involvement in the process of TPH depletion is putatively limited to intracellular pathways, since any of the *Firmicutes* harbored the DyP. In literature, *Planomicrobium* sp. has been described as capable to degrade alkanes (Radwan et al., 2019). *Bacillus* sp. is described for its capability to transform xenobiotics by the exploitation of intracellular and extracellular enzymatic machinery, associated to the capacity to produce biosurfactants (Arora, 2020).

## Conclusion

Our data showed that the mycoaugmentation approach was successful in both eliciting and accelerating the process of TPH depletion in a historically polluted soil, by establishing a metabolic network between bacteria and fungi. The soil autochthonous bacterial community played a pivotal role in the degradation of TPH by the involvement of both generalist and specialist species contributing to the process, in successive phases. The process of depletion catalyzed by the hydrocarbonoclastic specialists was primed by the adaptative strategy of the saprophytic generalists, which are reasonably involved in increasing the bioavailability of the carbon source that, in a historically polluted soil, is mainly consistent with the contamination. An increase in the abundance of *Ciboria* sp. has been pivotal to the acceleration of the onset of hydrocarbonoclastic specialist over generalist bacteria.

## Data Availability Statement

The datasets presentedy in this study can be found in online repositories. The names of the repository/repositories and accession number(s) can be found below: https://www.ncbi.nlm.nih.gov/, BioProject ID PRJNA688285; https://www.ncbi.nlm.nih.gov/, BioProject ID PRJNA688315; https://www.ncbi.nlm.nih.gov/, BioProject ID PRJNA688293.

## Author Contributions

SD designed the research, supervised the work, and wrote the manuscript. SB and IC participated in the design of the research, performed the laboratory experiments, bioinformatic analysis, and participated in the writing of the manuscript. SL performed the bioinformatic analysis and critically revised the manuscript. GS participated in the design of the research and performed the laboratory experiments. AB participated in the design of the research and critically revised the manuscript. GP and MG revised the manuscript. DL revised the design of the experiments and critically revised the manuscript. All the authors read and approved the manuscript and contributed to the article and approved the submitted version.

## Conflict of Interest

The authors declare that the research was conducted in the absence of any commercial or financial relationships that could be construed as a potential conflict of interest.

## Footnotes

1 https://web.rniapps.net/iVikodak/
