# Supplementary material for: A New Ciboria sp. for Soil Mycoremediation and the Bacterial Contribution to the Depletion of Total Petroleum Hydrocarbons
Source: Front Microbiol. 2021 Jun 8;12:647373. doi: 10.3389/fmicb.2021.647373 (PMC8221241; doi:10.3389/fmicb.2021.647373)
Supplement: Supplementary file 1 [file Data_Sheet_1.pdf]

Supplementary methods and results

ISO 16703:2004 (modified) method for Petroleum Hydrocarbons C_{10}-C_{40}.

Extraction and purification

Soil has been dried at room temperature for 5 days and dry weight was annotated. After drying, soil was manually grinded and sieved with 2 mm sieves.

20 gr of soil powder that passed 2 mm sieve, were liquid-extracted in Erlenmeyer flasks with glass sealed plugs, by mechanical shaking in orbital shaker at 120 rpm for 1 hour, in the dark and at room temperature.

The liquid extractant was n-heptane, containing n-decane ($C_{10}H_{22}$ 30 μL/L) and n-tetracontane ($C_{40}H_{82}$ 30 mg/L) mixed with acetone 1:2 v/v; 60 ml of this mixture were used every 20 gr of soil powder.

Extracted liquid phase was washed twice with 2 aliquots of H_2O (100 ml each) in a separatory funnel, and overlaying phase was recovered.

The purification step, constituted by adsorption of polar compounds on Florisil®, was skipped in order to quantify also extractable polar compounds like aromatic hydrocarbon derivatives.

Quantification of extracted organic compounds was performed by gas chromatography with Flame ionization detector (GC-FID).

Instrumental setup for CG FID

Injection volume: 1 μL

Injector: split-less mode, 300°C, septum purge 5 ml/min, injector purge flow 10 ml/min, by 10 minutes after injection.

Column: ZB 1 HT SCOT capillary column by Phenomenex, 15 m length, 0.25 mm internal diameter; 0,1 □m film thickness, carrier gas flow (He) 3.83 ml/min

Oven program: 60°C for 2 minutes, 40°C/min ramp to 350°C, 350°C for 2 minutes.

FID: 360°C, air flow 400 ml/min, hydrogen flow 40 ml/min, make-up flow (nitrogen) 30 ml/min sampling frequency 50 Hz.

Calibration

A calibration curve was performed by using dilutions in n-heptane of 8000 mg/L standard solution made by a 1:1 w/w mixing of mineral oil A and mineral oil B commercial standards (Sigma Aldrich).

Chromatogram integration was performed after subtraction of signal coming from an instrumental blank (n-heptane injection only), and only signal comprised between n-decane and n-tetracontane retention time was integrated, using corrected baseline as stated before.

The ratio between integrated area of the standard solutions and area of n-tetracontane peak (with natural baseline) was used to produce a calibration curve in function of standard concentration.

Method VIII.1 for Humic and Fulvic Acids quantification

10 g of air-dried and sieved soil were added to 250 ml Erlenmeyer flasks, containing 100 ml of 0.1 M sodium pyrophosphate and sodium hydroxide.

Solution was deaerated by nitrogen gas bubbling for a minute, plugged tightly with a rubber plug and extracted in a Dubnoff water-bath for 24 hours at 65 °C and 80 RPM.

Cold and decanted supernatant was centrifuged at 2700 rpm for 20 minutes and filtered at 0.45 mm on a cellulose-acetate membrane and put in a new centrifuge tube. Humic fraction was then precipitated by addition of sulfuric acid 50% v.v until pH dropped below 2, and the centrifugation step was repeated. Residual supernatant was then cleaned up by SPE partition

10 ml polypropylene chromatographic columns for SPE separation were loaded with approximately 5 g of polyvinylpyrrolidone resin slurry, previously hydrated and conditioned to pH 1.5 by sulfuric acid 0.005 M.

The supernatant was loaded in SPE column and washed with 25 ml of sulfuric acid 0.005 M. Then eluent was exchanged with sodium hydroxide 0.5 M. The flowthrough was discarded until first brown drops (fulvic acids) came out, and collected until no colouring was spotted. The solid fraction achieved in the first step of precipitation (humic acids) and the eluate from SPE step were merged, and final volume was adjusted at 50 ml with sodium hydroxide solution 0.5 M.

Digestion and titration

10 ml of the merged solution were transferred in a digestion tube with 20 ml of potassium bichromate 0.3334 M, 26 ml of sulfuric acid 96% and some crystals of sliver sulphate, and quickly heated at 160 °C. The reaction was stopped after 10 minutes by dilution to 200 ml with cold water.

20 ml of resulting solution were added with 100 ml of water, 8 ml of H_3PO_4 85% and 0.5 ml of redox indicator (4-diphenylamino sulfonate sodium salt in concentrated sulfuric acid) and titrated with a solution of $FeSO_4$ 0.2M

SUPPLEMENTARY RESULTS

two-way RM ANOVA for TPH and HFA

Here we report detailed statistic tables for two-way RM ANOVA performed on TPH concentration and HFA concentration:

Table S1: statistical report of two-way RM ANOVA for TPH and HFA. P-values > 0.05 are considered not significant

two-way RM ANOVA	TPH		HFA	
Source of Variation	% of total variation	P-value	% of total variation	P-value
Interaction	37.72	< 0.0001	29.07	0.0138
Days	41.00	< 0.0001	29.66	0.0017
Treatment	19.98	< 0.0001	9.185	0.1154
Subjects (matching)	0.2102	0.7385	8.713	0.3906

In both TPH and HFA, non-significance of subject matching underlines that the assumption that samples are paired within days variable can be discarded (i.e. 30 days interval is enough to consider samples datapoints belonging to the same time series as independent).

Form this result, we assumed that subsequent statistical tests for significance that take into account independency between datapoints could be chosen, with the advantage of an increased statistical power.

Below we reported the multiple comparisons from Tuckey post hoc test for TPH quantification analysis. In the table, comparisons within each condition among the different sampling times are reported. For each comparison p-values are reported.

Table S2: Tuckey multiple comparison test for TPH quantification data.

Tukey's multiple comparisons test	Mean Diff,	95% CI of diff,	Significant?	Summary	Adjusted P Value
CNT					
0 vs. 30	932.7	-1419 to 3285	No	ns	0.6965
0 vs. 60	-1222	-3574 to 1130	No	ns	0.4915
0 vs. 90	512.3	-1840 to 2864	No	ns	0.9308
30 vs. 60	-2155	-4507 to 196,9	No	ns	0.0808
30 vs. 90	-420.3	-2772 to 1932	No	ns	0.9599
60 vs. 90	1735	-617,2 to 4087	No	ns	0.2036
F1					
0 vs. 30	637.3	-1715 to 2989	No	ns	0.8768
0 vs. 60	-305	-2657 to 2047	No	ns	0.9839
0 vs. 90	5016	2664 to 7368	Yes	****	< 0,0001
30 vs. 60	-942.3	-3294 to 1410	No	ns	0.6898
30 vs. 90	4379	2027 to 6731	Yes	***	0.0002
60 vs. 90	5321	2969 to 7673	Yes	****	< 0,0001
F7					
0 vs. 30	-1697	-4049 to 654,6	No	ns	0.2193
0 vs. 60	6003	3651 to 8355	Yes	****	< 0,0001
0 vs. 90	8102	5750 to 10454	Yes	****	< 0,0001
30 vs. 60	7701	5349 to 10053	Yes	****	< 0,0001
30 vs. 90	9800	7448 to 12152	Yes	****	< 0,0001
60 vs. 90	2099	-252,9 to 4451	No	ns	0.0922

Below we reported the multiple comparisons from Tuckey post hoc test for HFA quantification analysis. In the table comparisons within each condition among the different sampling times are reported. For each comparison p-values are reported.

Table S3: Tuckey multiple comparison test for HFA quantification data.

Tukey's multiple comparisons test	Mean Diff,	95% CI of diff,	Significant?	Summary	Adjusted P Value
CNT					
0 vs. 30	-1.233	-7,780 to 5,314	No	ns	0.95
0 vs. 60	-0.3	-6,847 to 6,247	No	ns	0.9992
0 vs. 90	-0.1367	-6,684 to 6,410	No	ns	> 0,9999
30 vs. 60	0.9333	-5,614 to 7,480	No	ns	0.9772
30 vs. 90	1.097	-5,450 to 7,644	No	ns	0.964
60 vs. 90	0.1633	-6,384 to 6,710	No	ns	0.9999
F1					
0 vs. 30	6.2	-0,3470 to 12,75	No	ns	0.0671
0 vs. 60	-0.4667	-7,014 to 6,080	No	ns	0.997
0 vs. 90	-5.867	-12,41 to 0,6804	No	ns	0.0883
30 vs. 60	-6.667	-13,21 to -0,1196	Yes	*	0.0451
30 vs. 90	-12.07	-18,61 to -5,520	Yes	***	0.0003
60 vs. 90	-5.4	-11,95 to 1,147	No	ns	0.1279
F7					
0 vs. 30	-2.667	-9,214 to 3,880	No	ns	0.6638
0 vs. 60	-6.1	-12,65 to 0,4470	No	ns	0.0729
0 vs. 90	-9.1	-15,65 to -2,553	Yes	**	0.005
30 vs. 60	-3.433	-9,980 to 3,114	No	ns	0.468
30 vs. 90	-6.433	-12,98 to 0,1137	No	ns	0.0551
60 vs. 90	-3	-9,547 to 3,547	No	ns	0.5776

CCA analysis for bacterial and fungal community

CCA2 axes in both figure 3 panel B and figure 5 panel B represent a very low amount of inertia. In Table S2 results produced by anova.cca (data = X, model = "full", by = "axis") script string are reported for each dataset.

Table S4: PERMANOVA test for axis significance. *** = p-value < 0.001, n.s. = not significative

Permutation test for CCA under full model (999 repetitions)					
Model:	ASV ~ TPH + HFA				
Bacterial community					
	Degrees of freedom	ChiSquare	F	Pr(>F)	
CCA1	1	0.19643	14.8984	0.001	***
CCA2	1	0.00856	0.6494	0.779	n.s.
Residual	33	0.4351			
Fungal community					
	Degrees of freedom	ChiSquare	F	Pr(>F)	
CCA1	1	0.39524	15.524	0.001	***
CCA2	1	0.00422	0.1656	0.894	n.s.
Residual	33	0.84018			

This evidences also that HFA projection on CCA2 axis in figure 3 panel B and figure 5 panel B is neglectable.

Even if a significative variance in HFA content during mesocosms experiments can be observed in figure 1 at least for F7 mesocosm, is worth to evaluate if HFA constrained variable influences bacterial composition in samples significatively.

Subsequent statistical analysis of significance for constrained variables were performed as described for axis significance, by anova.cca(data = X, model = "full", by = "terms") script string.

Table S5: PERMANOVA test for unconstrained variables significance. *** = p-value < 0.001, n.s. = not significative

Permutation test for CCA under full model					
Model:	ASV ~ TPH + HFA				
Bacterial community					
	Degrees of freedom	ChiSquare	F	Pr(>F)	
TPH	1	0.19187	14.5523	0.001	***
HFA	1	0.01313	0.9956	0.377	
Residual	33	0.4351			
Fungal community					
	Degrees of freedom	ChiSquare	F	Pr(>F)	
TPH	1	0.39067	15.345	0.001	***
HFA	1	0.00878	0.345	0.715	
Residual	33	0.84018			

Table S5 shows that inertia shown is represented significatively only by TPH term (p-value ≤ 0.001).

Same result is observed for CCA analysis performed on fungal community.

SUPPLEMENTARY FIGURES

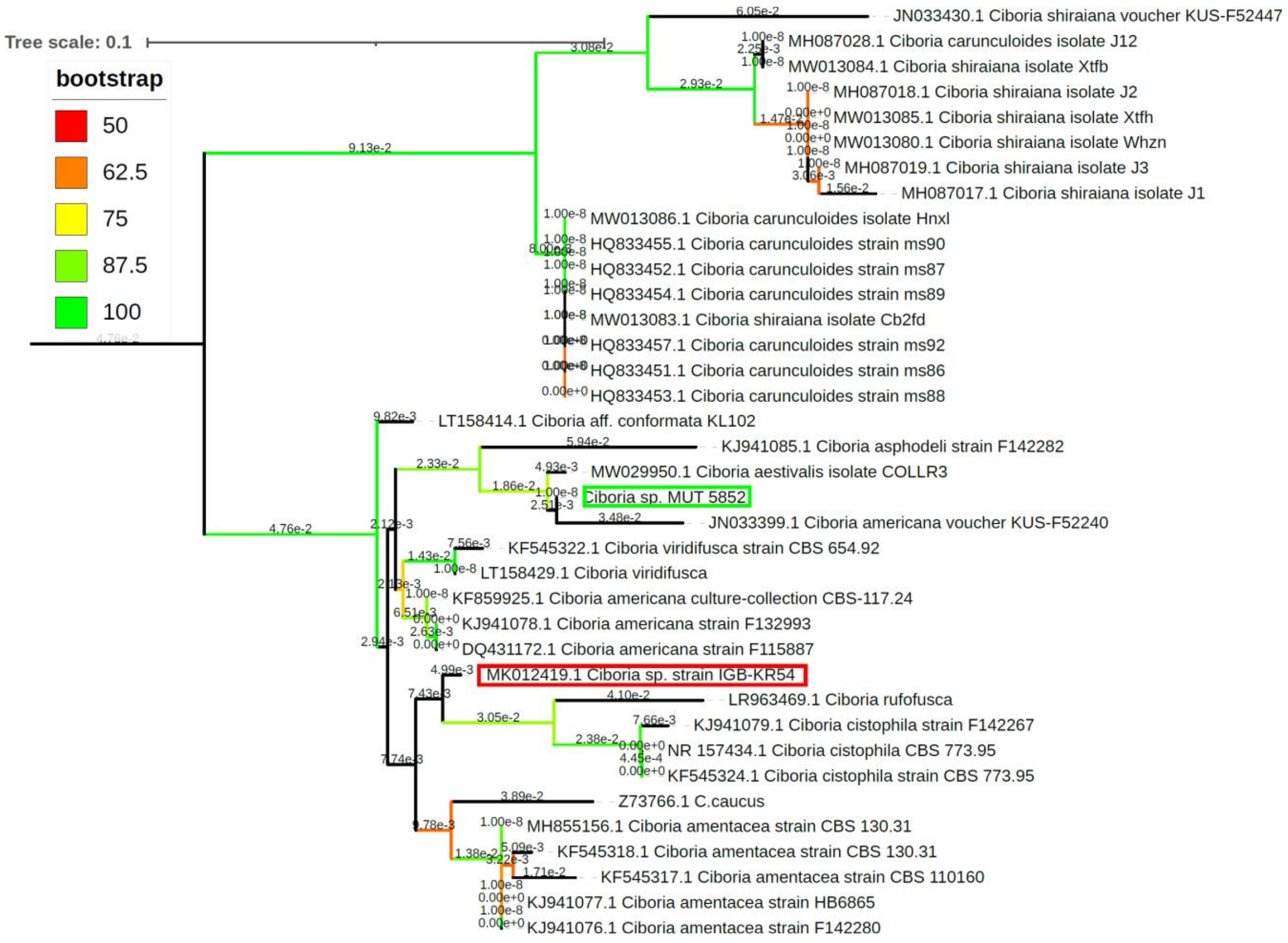

Figure S1: Phylogenetic tree of *Ciboria* sp.: ITS sequences where retrieved from NCBI under accession number reported on each tip. Sequences where aligned, staggered and masked with DECIPHER package (v 2.18.1), using default settings. Phylogenetic Maximum Likelihood Tree was performed by Phangorn package (v 2.5.5). GRT model, with four Gamma distributions and invariant sites, was used to infer the initial tree. A bootstrapping process with 1000 repetition was performed to evaluate node robustness. Branch colours represent bootstrapping values for subsequent nodes. Numeric values reported over branches represent their length. Green rectangle encloses *Ciboria* sp. MUT 5852, while red rectangle encloses *Ciboria* sp. reported to degrade Azo-dies (Perkins *et al.* 2019)

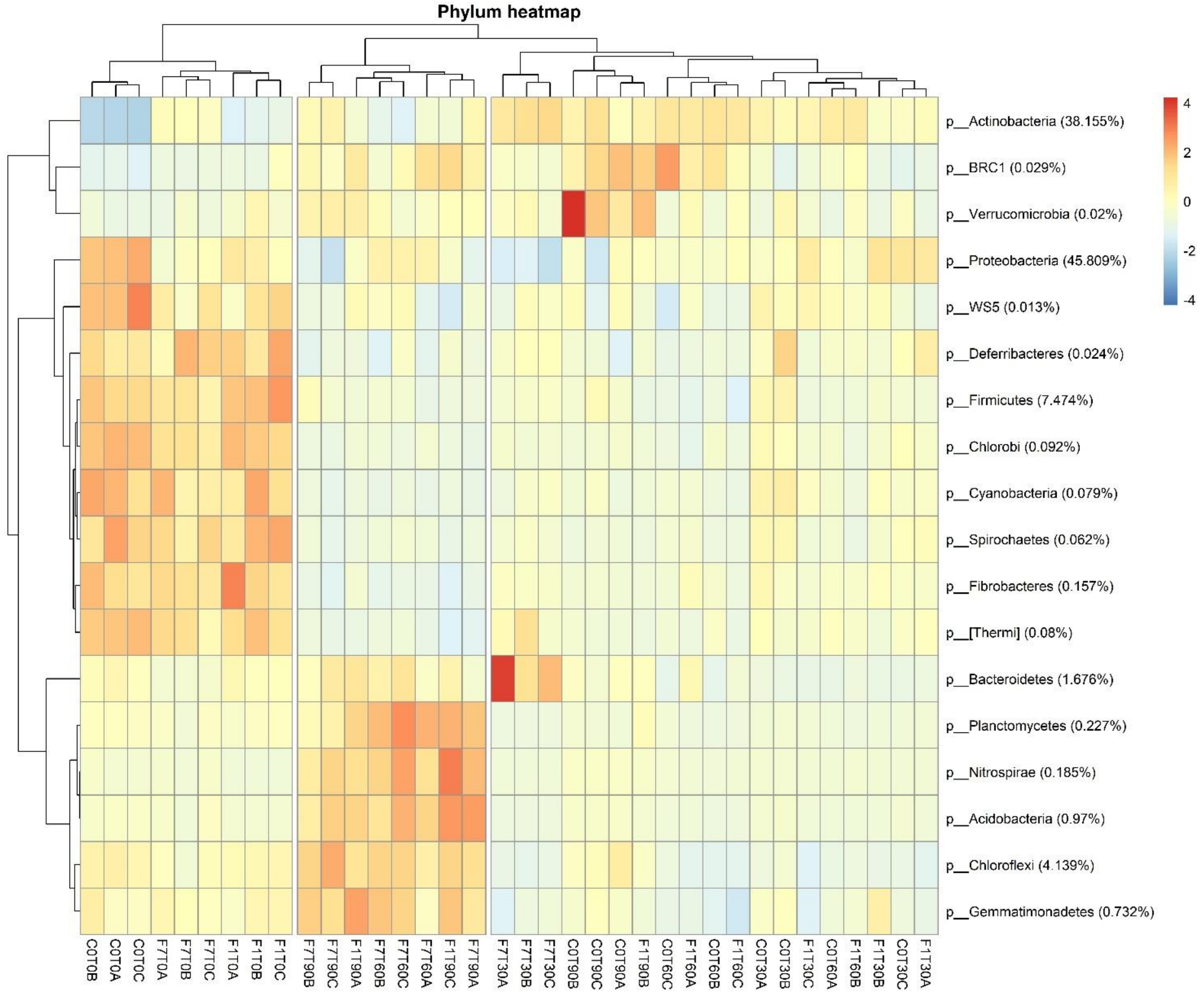

Figure S2: Heatmap showing bacterial ASV abundances per sample at phylum level with a cut-off of 0.01%. Hierarchical clustering was performed on both rows and columns by Pearson correlation, based on Euclidean distance. Colour scheme represents row-wise Z-scores of ASV counts per ASV. Percentage reported near ASV names represent the relative abundance of the sum of ASV counts per sample against total sum (i.e. Z=0 matches reported percentage).

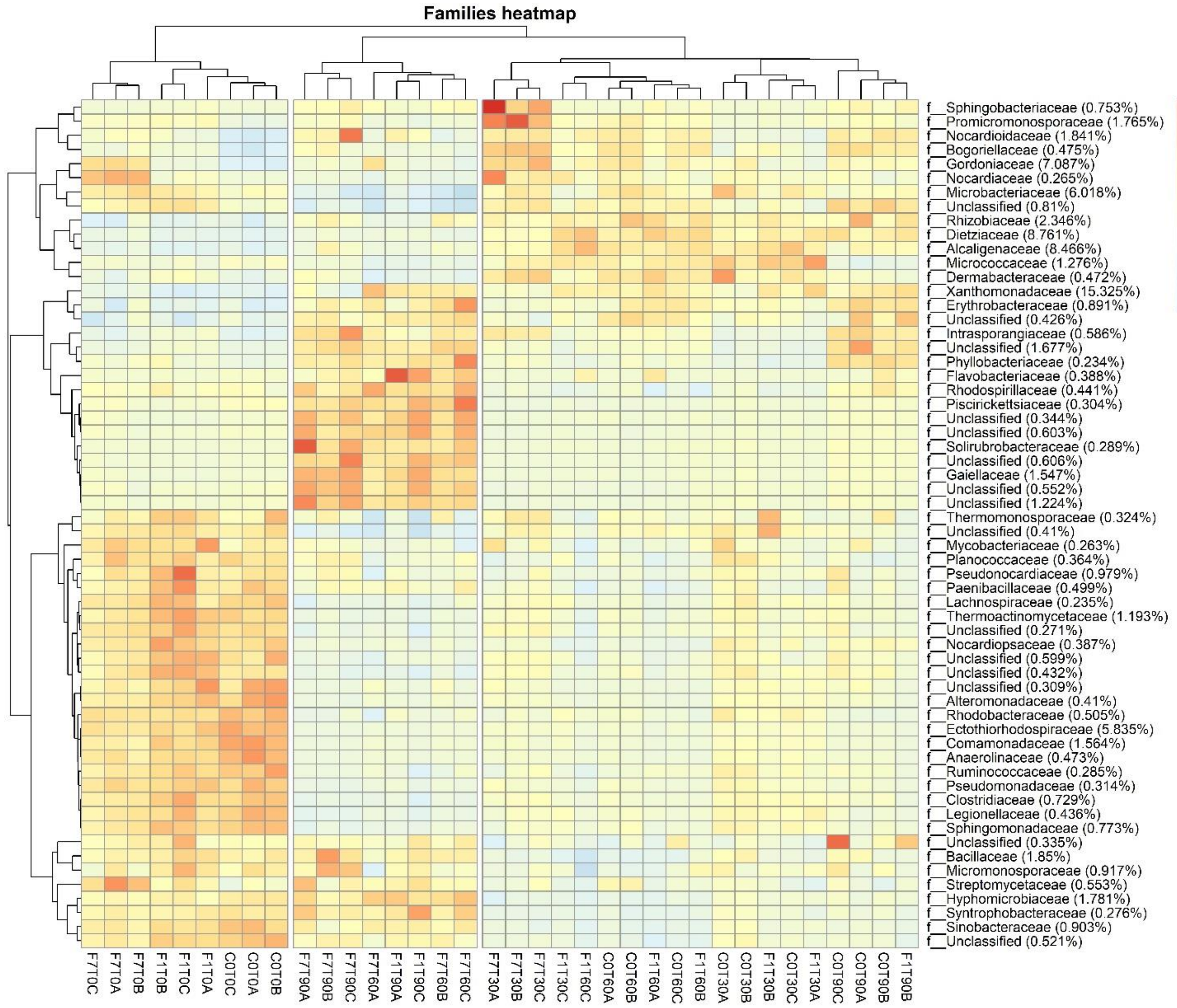

Figure S3: Heatmaps showing bacterial ASV abundances per sample at family level with a cut-off of 0.1%. Hierarchical clustering was performed on both rows and columns by Pearson correlation, based on Euclidean distance. Colour scheme represents row-wise Z-scores of ASV counts per ASV. Percentage reported near ASV names represent the relative abundance of the sum of ASV counts per sample against total sum (i.e. Z=0 matches reported percentage). Unclassified groups were not pooled.

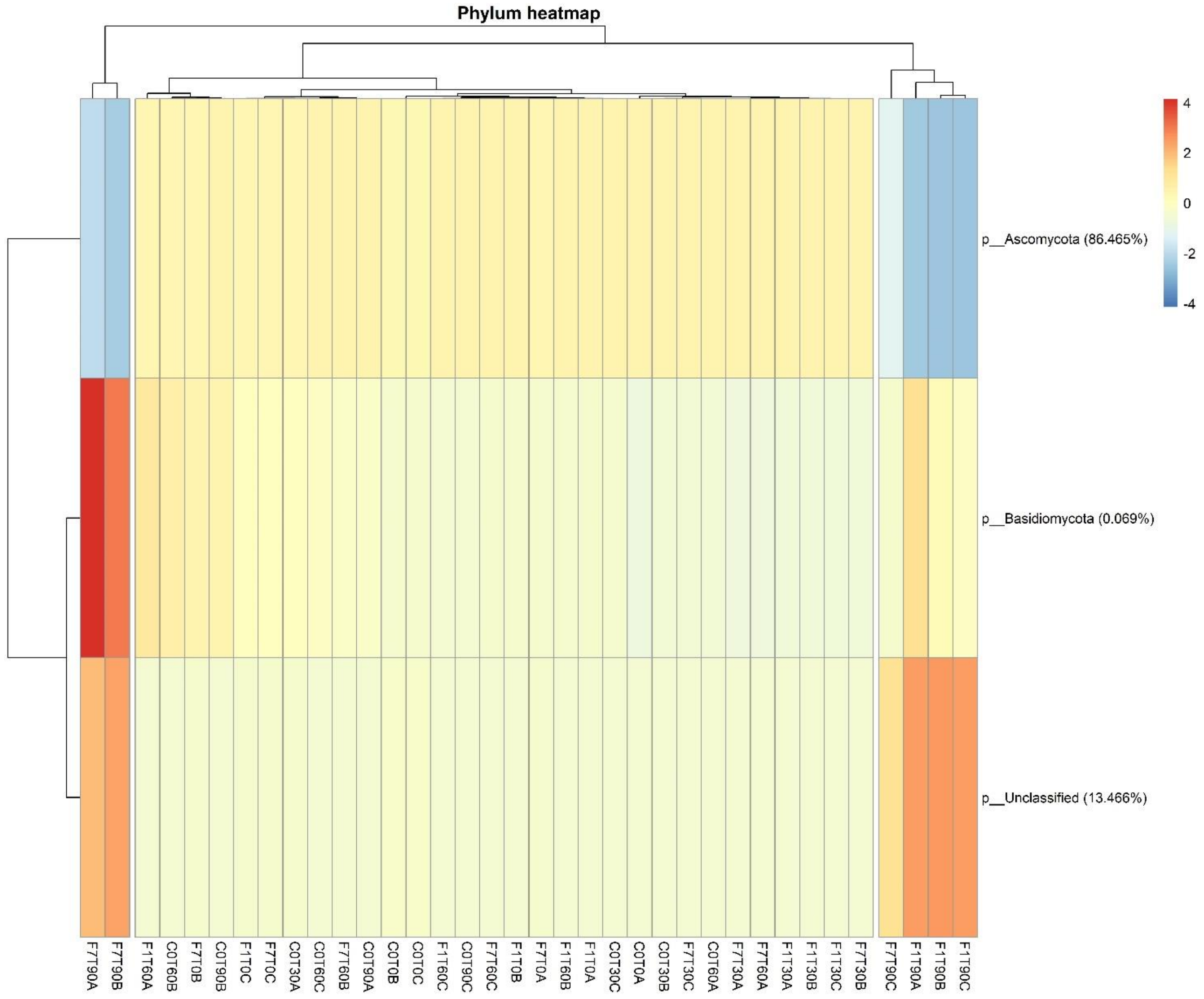

Figure S4: Heatmaps showing fungal ASV abundances per sample at phylum level with a cut-off of 0.01%. Hierarchical clustering was performed on both rows and columns by Pearson correlation, based on Euclidean distance. Colour scheme represents row-wise Z-scores of ASV counts per sample. Percentage reported near ASV names represent the relative abundance of the sum of ASV counts per sample against total sum (i.e. Z=0 matches reported percentage).

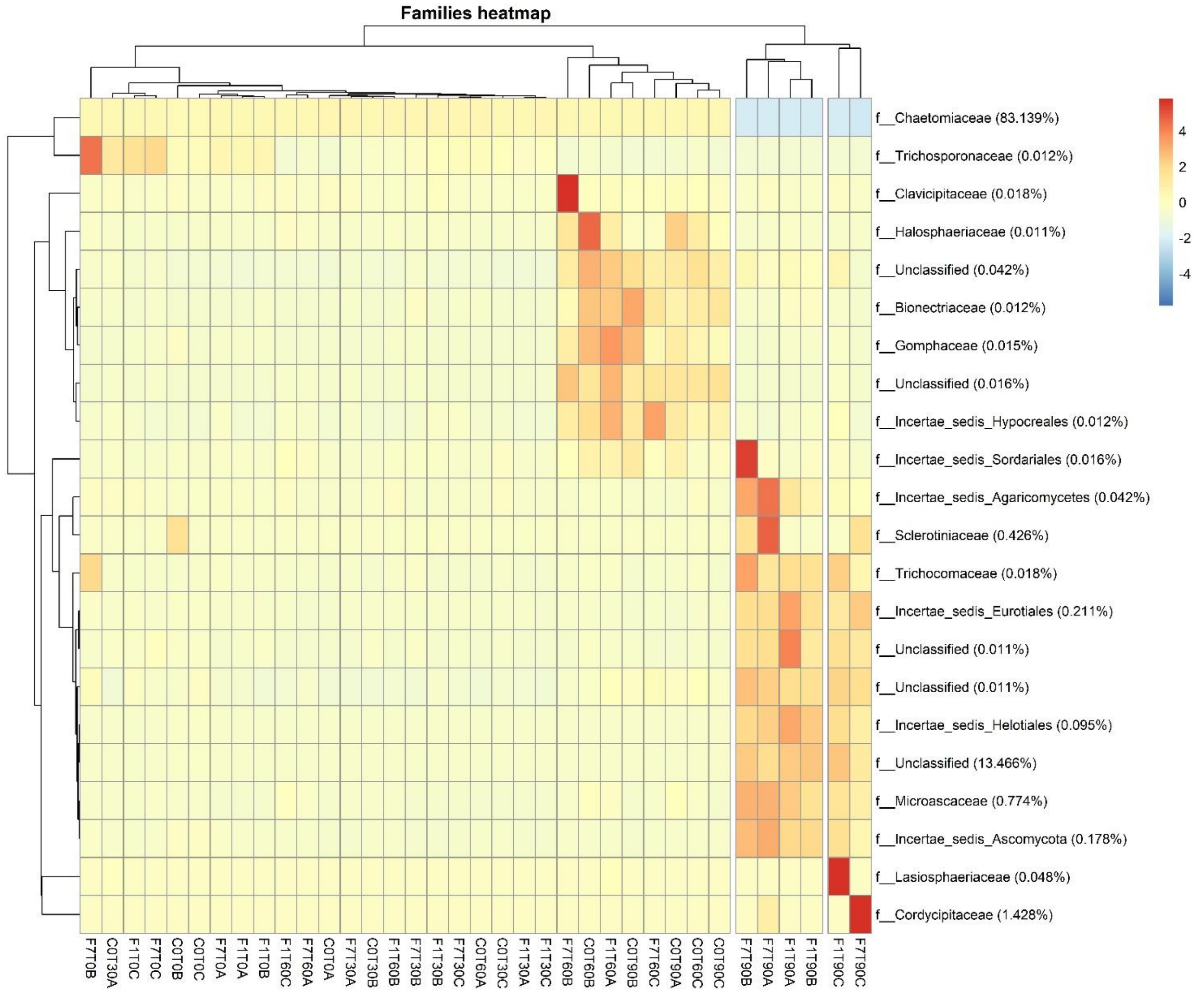

Figure S5: Heatmaps showing fungal ASV abundances per sample at family level with a cut-off of 0.01%. Hierarchical clustering was performed on both rows and columns by Pearson correlation, based on Euclidean distance. Colour scheme represents row-wise Z-scores of ASV counts. Percentage reported near ASV names represent the relative abundance of the sum of ASV counts per sample against total sum (i.e. Z=0 matches reported percentage). Unclassified groups were not pooled.

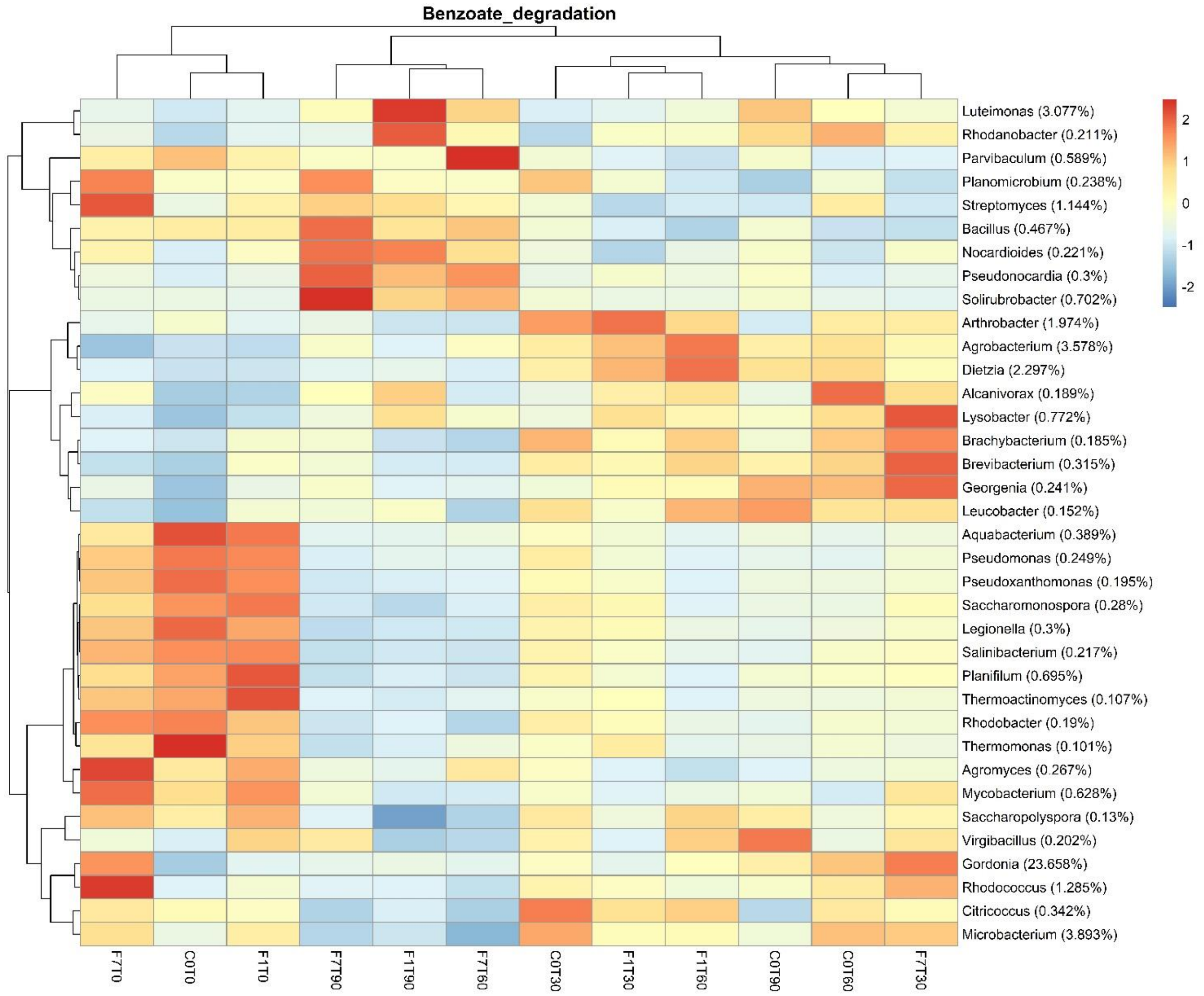

Figure S6: Heatmap showing Bacterial contributions to Benzoate degradation pathway in metagenome, as calculated by iVikodak server, with a PEC of 80%. Reported taxonomic resolution is genus level, with a cut-off of 0.1%. Hierarchical clustering was performed on both rows and columns by Pearson correlation, based on Euclidean distance. Colour scheme represents row-wise Z-scores of contribution counts per ASV. Percentage reported near ASV names represent the relative abundance of the sum of ASV contributions per sample against total sum (i.e. Z=0 matches reported percentage).

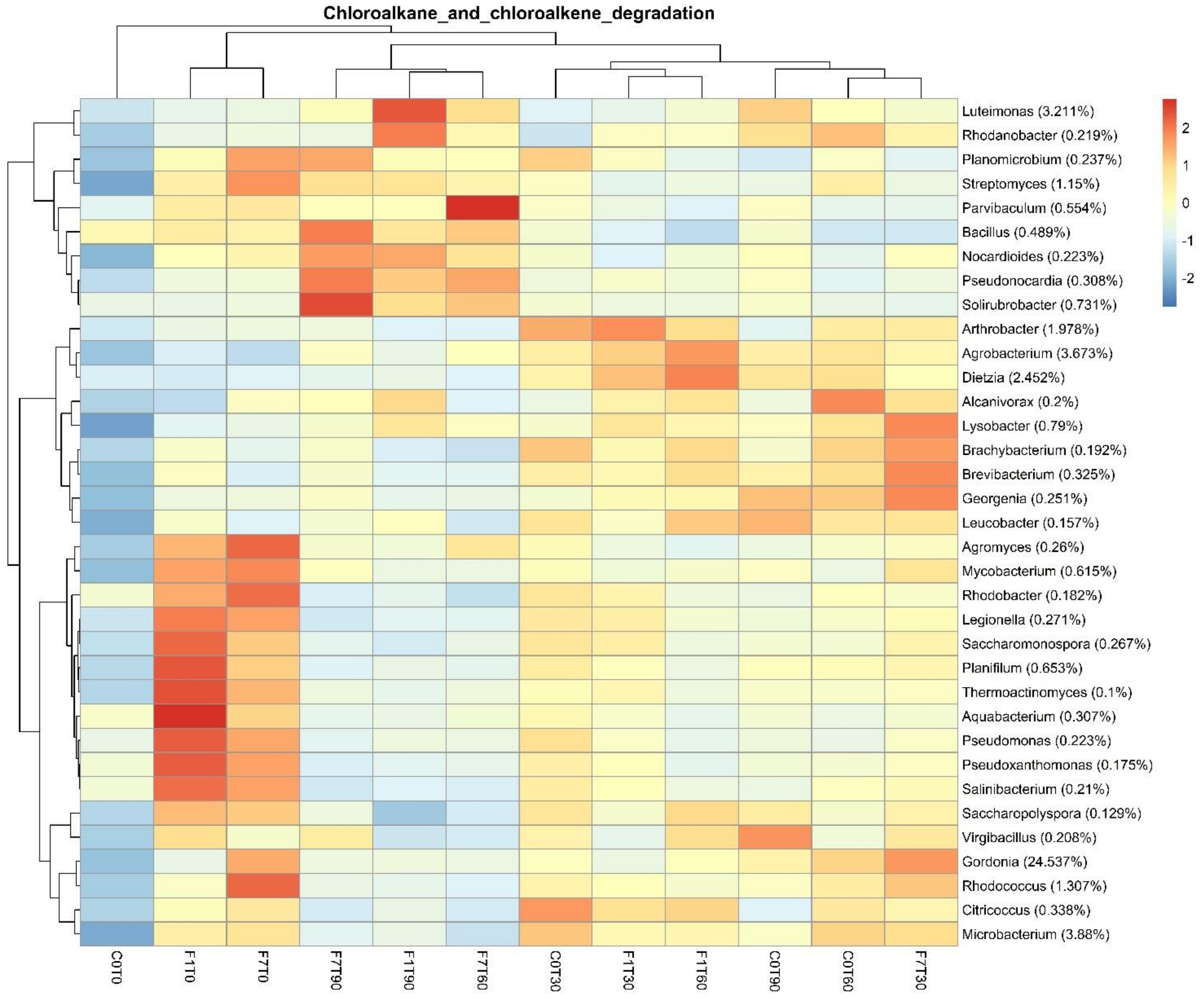

Figure S7: Heatmap showing Bacterial contributions to Chloroalkane and Chloroalkene degradation pathway in metagenome, as calculated by iVikodak server, with a PEC of 80%. Reported taxonomic resolution is genus level, with a cut-off of 0.1%. Hierarchical clustering was performed on both rows and columns by Pearson correlation, based on Euclidean distance. Colour scheme represents row-wise Z-scores of contribution counts per ASV. Percentage reported near ASV names represent the relative abundance of the sum of ASV contributions per sample against total sum (i.e. Z=0 matches reported percentage).

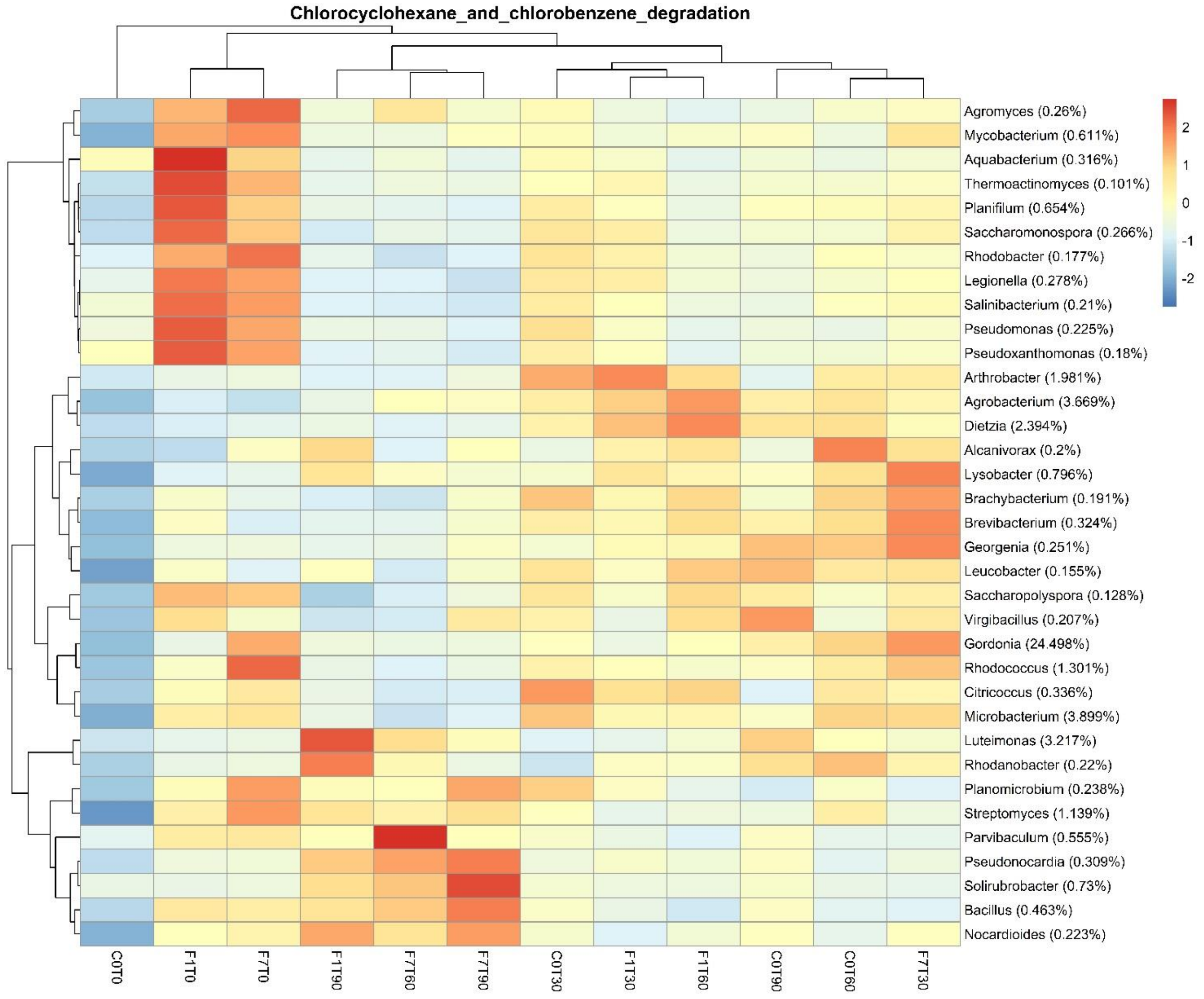

Figure S8: Heatmap showing Bacterial contributions to Chlorociclohexane and Chlorobenzene degradation pathway in metagenome, as calculated by iVikodak server, with a PEC of 80%. Reported taxonomic resolution is genus level, with a cut-off of 0.1%. Hierarchical clustering was performed on both rows and columns by Pearson correlation, based on Euclidean distance. Colour scheme represents row-wise Z-scores of contribution counts per ASV. Percentage reported near ASV names represent the relative abundance of the sum of ASV contributions per sample against total sum (i.e. Z=0 matches reported percentage).

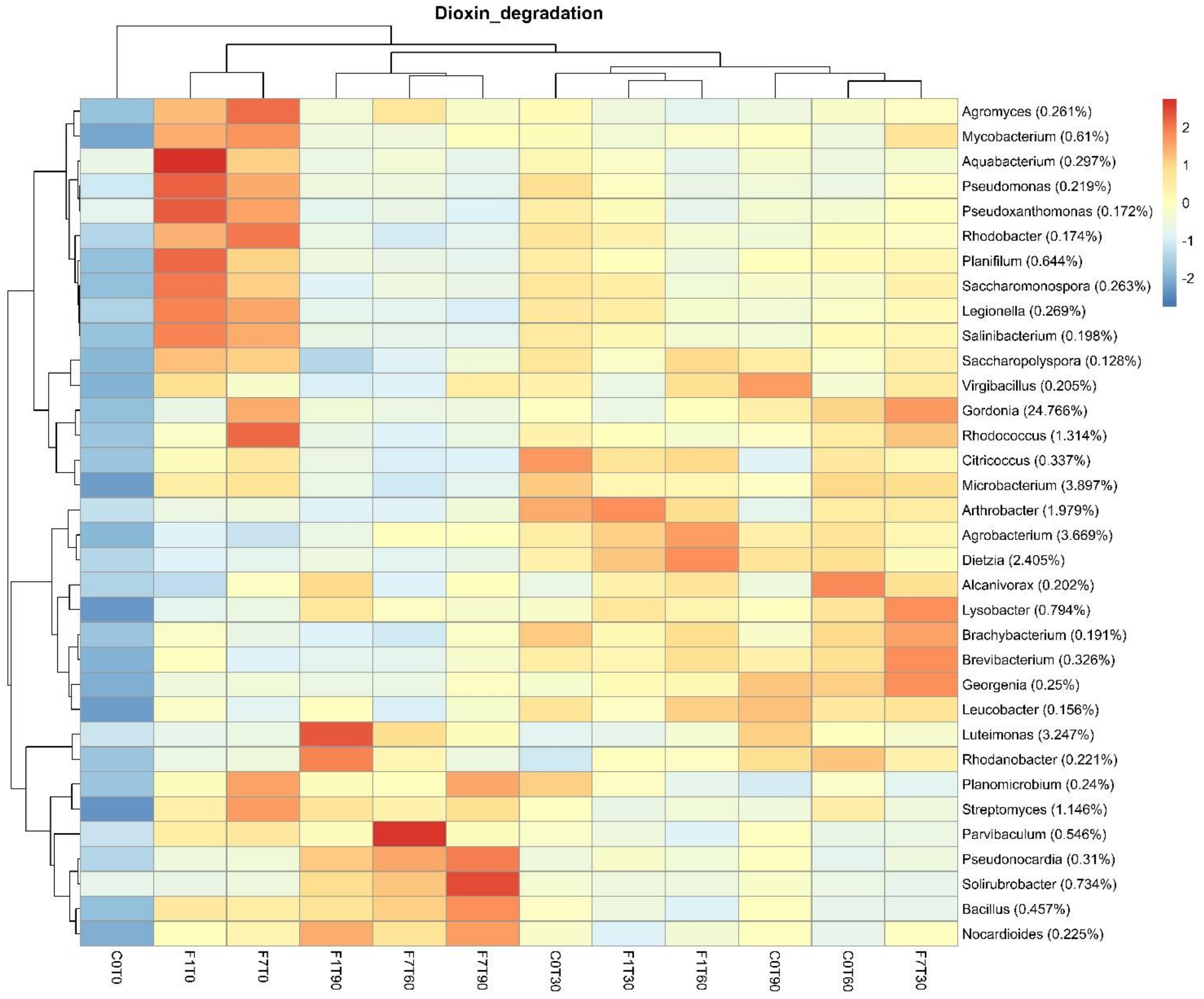

Figure S9: Heatmap showing Bacterial contributions to Dioxin degradation pathway in metagenome, as calculated by iVikodak server, with a PEC of 80%. Reported taxonomic resolution is genus level, with a cut-off of 0.1%. Hierarchical clustering was performed on both rows and columns by Pearson correlation, based on Euclidean distance. Colour scheme represents row-wise Z-scores of contribution counts per ASV. Percentage reported near ASV names represent the relative abundance of the sum of ASV contributions per sample against total sum (i.e. Z=0 matches reported percentage).

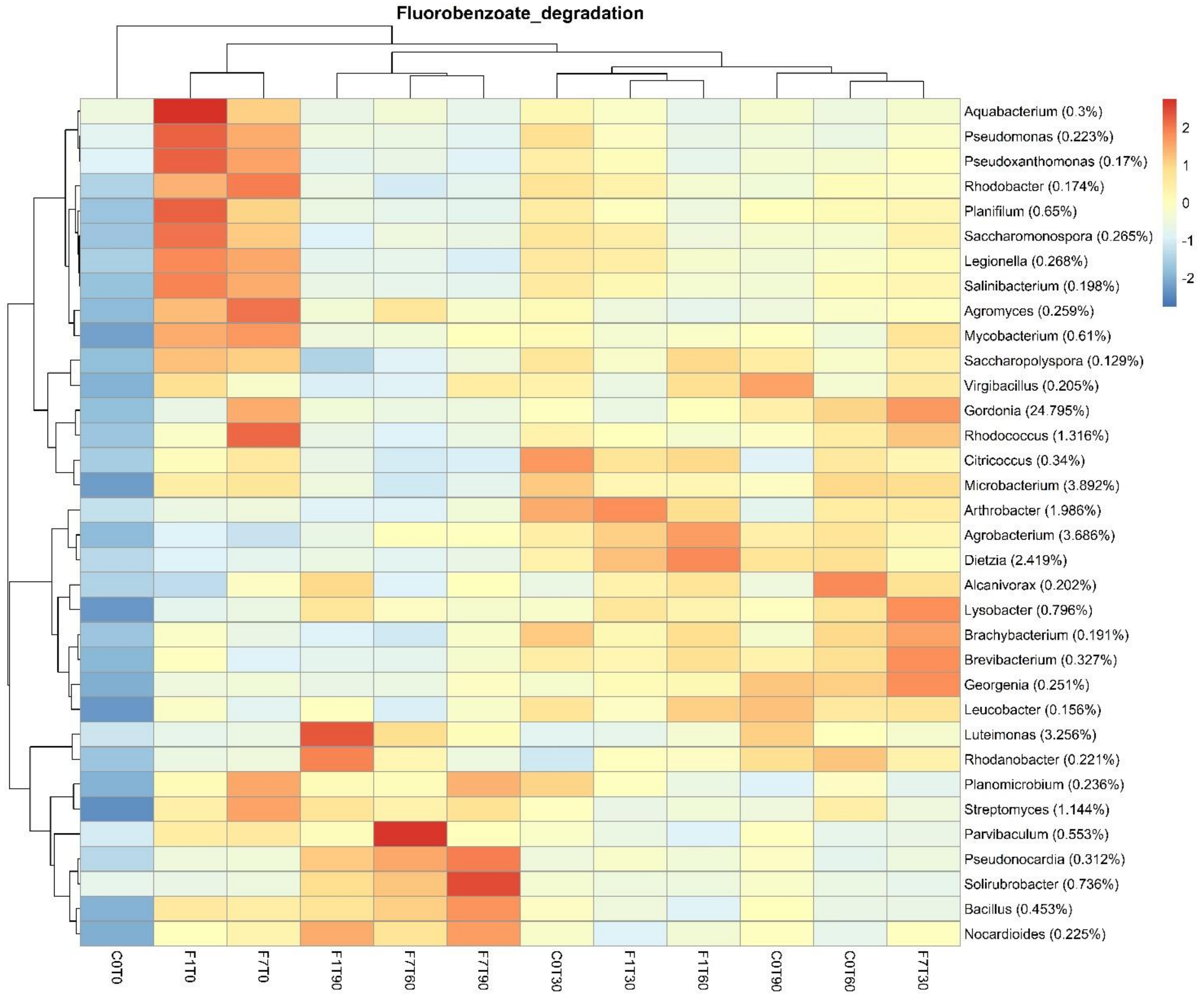

Figure S10: Heatmap showing Bacterial contributions to Fluoroenzoate degradation pathway in metagenome, as calculated by iVikodak server, with a PEC of 80%. Reported taxonomic resolution is genus level, with a cut-off of 0.1%. Hierarchical clustering was performed on both rows and columns by Pearson correlation, based on Euclidean distance. Colour scheme represents row-wise Z-scores of contribution counts per ASV. Percentage reported near ASV names represent the relative abundance of the sum of ASV contributions per sample against total sum (i.e. Z=0 matches reported percentage).

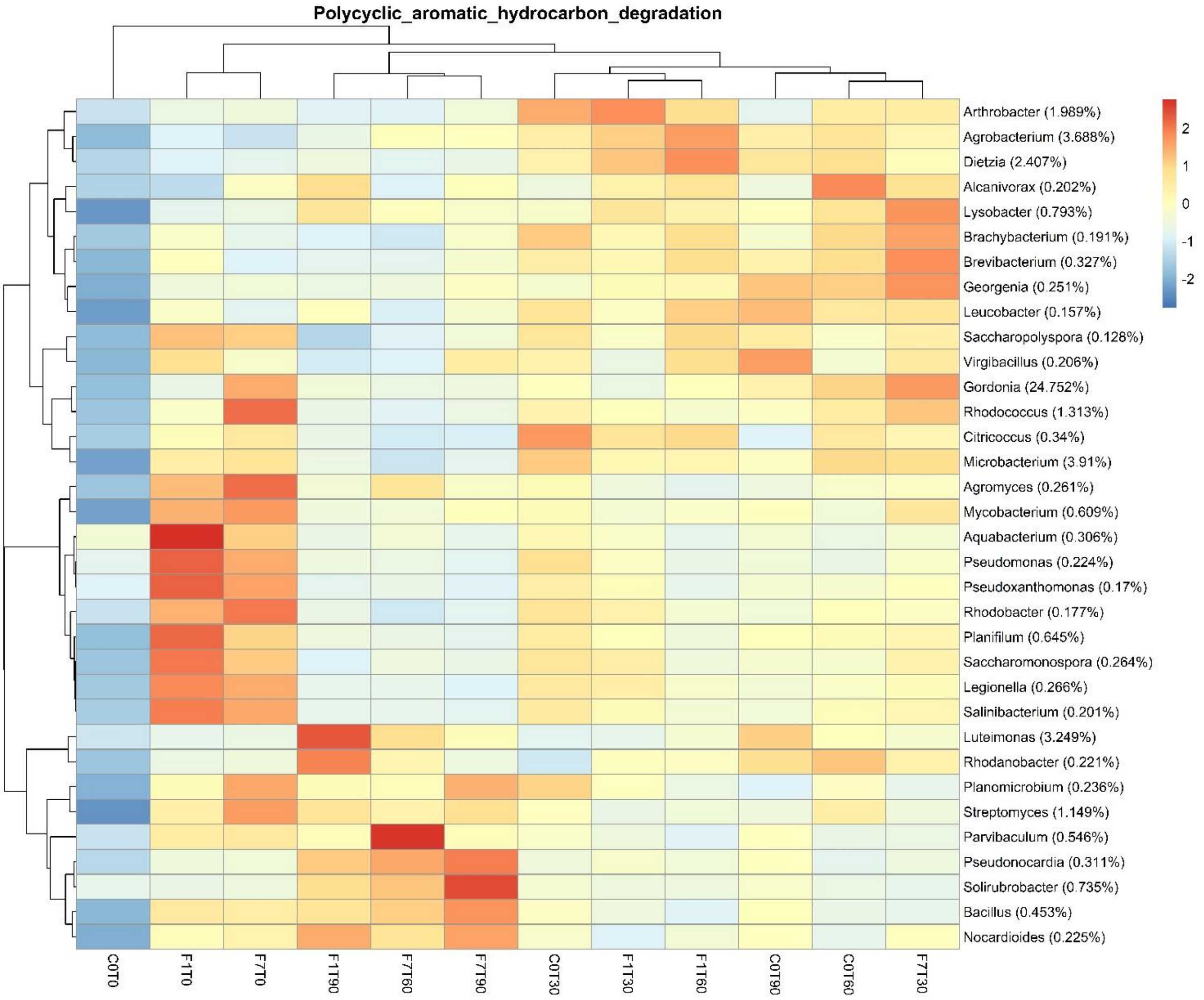

Figure S11: Heatmap showing Bacterial contributions to Polycyclic aromatic hydrocarbon degradation pathway in metagenome, as calculated by iVikodak server, with a PEC of 80%. Reported taxonomic resolution is genus level, with a cut-off of 0.1%. Hierarchical clustering was performed on both rows and columns by Pearson correlation, based on Euclidean distance. Colour scheme represents row-wise Z-scores of contribution counts per ASV. Percentage reported near ASV names represent the relative abundance of the sum of ASV contributions per sample against total sum (i.e. Z=0 matches reported percentage).

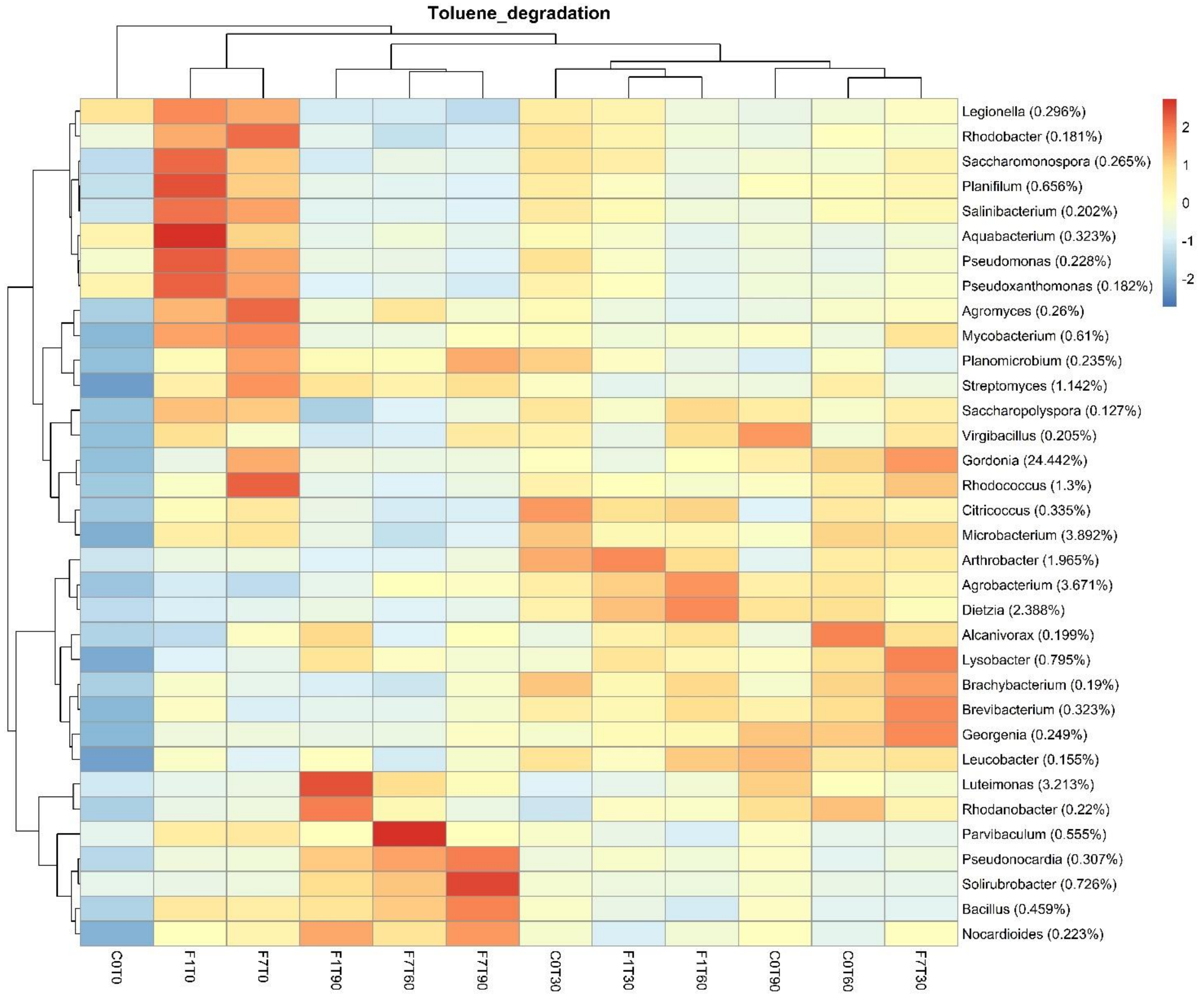

Figure S12: Heatmap showing Bacterial contributions to Toluene degradation pathway in metagenome, as calculated by iVikodak server, with a PEC of 80%. Reported taxonomic resolution is genus level, with a cut-off of 0.1%. Hierarchical clustering was performed on both rows and columns by Pearson correlation, based on Euclidean distance. Colour scheme represents row-wise Z-scores of contribution counts per ASV. Percentage reported near ASV names represent the relative abundance of the sum of ASV contributions per sample against total sum (i.e. Z=0 matches reported percentage).

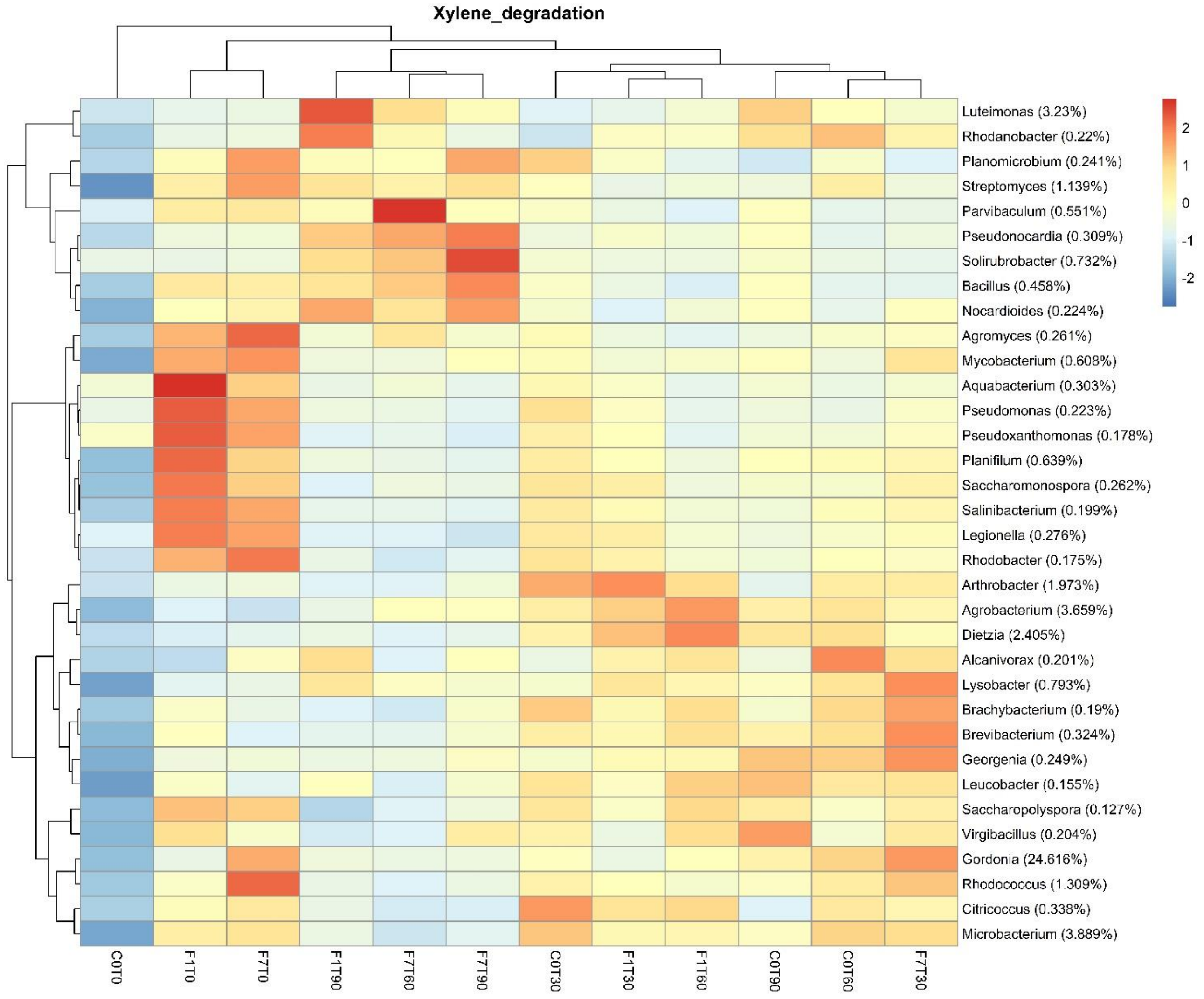

Figure S13: Heatmap showing Bacterial contributions to Xylene degradation pathway in metagenome, as calculated by iVikodak server, with a PEC of 80%. Reported taxonomic resolution is genus level, with a cut-off of 0.1%. Hierarchical clustering was performed on both rows and columns by Pearson correlation, based on Euclidean distance. Colour scheme represents row-wise Z-scores of contribution counts per ASV. Percentage reported near ASV names represent the relative abundance of the sum of ASV contributions per sample against total sum (i.e. Z=0 matches reported percentage).
